# Supplementary material for: Does information structuring improve recall of discharge information? A cluster randomized clinical trial
Source: PLoS One. 2021 Oct 18;16(10):e0257656. doi: 10.1371/journal.pone.0257656 (PMC8523048; doi:10.1371/journal.pone.0257656)
Supplement: S1 File — (DOCX) [file pone.0257656.s002.docx]

**Does Information Structuring Improve Recall of Discharge Information? A Cluster Randomized Clinical Trial**

Victoria Siegrist, Rui Mata, Wolf Langewitz, Heike Gerger, Stephan Furger, Ralph Hertwig, Roland Bingisser

**S1 File. Content of the communication training**

i) The “*general communication with ED patients*”-module was standard practice at the ED for new physicians, embedded in lectures focusing on principles of triage, working with protocols, legal aspects, and the most important patient needs. Discharge information was one of the main topics, focusing on the needs of junior physicians. The results of recent studies^10,36,37^ were discussed, emphasizing the content of discharge information, namely: diagnosis, follow-up, advice on self-care, red flags, and treatment.

ii) The “*empathy or structure skills*”-module covered the following topics in both groups of physicians: evidence from communication research, teaching of communication skills, and practical training in groups of two. Thereafter, the study protocol was explained, informed consent obtained, and surveys on demographics and physicians’ understanding on the acquired communication skills (scored on a numeric rating scale (NRS), range 0-10) were completed. Physicians in the E group were taught to respond to patients’ emotions with the tool NURSE.^38^ The acronym N-U-R-S-E assists with responding verbally to patients’ emotions: **N**ame the emotion; **U**nderstand the emotion; **R**espect the patient; **S**upport the patient; **E**xplore the emotion. Physicians in the S group were taught to use explicit verbal structuring, by (1) presenting discharge information on the basis of the “book metaphor”,^14,15,16^ and (2) selecting information according to the acronym InFARcT^36^. The book metaphor describes an advance organizer, whereas the acronym InFARcT defines relevant topics to be addressed in any discharge communication (i.e., **In**formation on diagnosis, **F**ollow-up, **A**dvice on self-care, **R**ed flags, **c**omplete **T**reatment).

iii) The “*feedback on the job*”-module consisted of an individual feedback on the job. As physicians were discharging a patient, a communication expert accompanied them. After being introduced, the expert remained silent during discharge communication. Subsequently, the physician received feedback pertaining to the E or S focus of his training.
