# Supplementary material for: Does information structuring improve recall of discharge information? A cluster randomized clinical trial
Source: PLoS One. 2021 Oct 18;16(10):e0257656. doi: 10.1371/journal.pone.0257656 (PMC8523048; doi:10.1371/journal.pone.0257656)
Supplement: S1 Protocol — (DOCX) [file pone.0257656.s003.docx]

Clinical Study Protocol

Improving Discharge Communication in the Emergency Department through Information Structuring: A Cluster Randomized Controlled Trial

**(BACOP)**

| Study Type: | Clinical trial with Investigational Medicinal Product (IMP), Medical Device (MD) |
| --- | --- |
| Study Categorisation: | A |
| Study Registration: | The study will be registered as soon as the ethics committee tells us to register. |
| Study Identifier: | NA |
| Sponsor-Investigator: | Prof. Dr. Roland Bingisser  Department of Emergency Medicine  University Hospital of Basel  Petersgraben 2  CH-4031 Basel  Switzerland  Mail: roland.bingisser@ubs.ch  Phone: +41 61 265 58 30 |
| Investigational Product: | Communication skills training |
| Protocol Version and Date: | 02, 02/01/2015 |

| Study number | EKNZ 2014-379 |
| --- | --- |
| Study Title | Improving Discharge Communication in the Emergency Department through Information Structuring: A Cluster Randomized Controlled Trial |

The Sponsor-Investigator has approved the protocol version 2, January 2, 2015, and confirms hereby to conduct the study according to the protocol, current version of the World Medical Association Declaration of Helsinki, ICH-GCP guidelines or ISO 14155 norm if applicable and the local legally applicable requirements.

Sponsor-Investigator:

Prof. Dr. Roland Bingisser

Basel, 02 of January 2015

| Place/Date |  |  |
| --- | --- | --- |
|  |  |  |

Local Principal Investigator at study site:

I have read and understood this trial protocol and agree to conduct the trial as set out in this study protocol, the current version of the World Medical Association Declaration of Helsinki, ICH-GCP guidelines or ISO 14155 norm and the local legally applicable requirements.

| Site | University Hospital of Basel  Petersgraben 2  4031 Basel  Switzerland |
| --- | --- |
| Principal investigator: |  |

Prof. Dr. Roland Bingisser

Basel^,^ 02 of January 2015

| Place/Date |  |
| --- | --- |

Table of Contents

Study synopsis 8

study summary in local language 13

Study schedule 14

1. STUDY ADMINISTRATIVE STRUCTURE 15

1.1 Sponsor, Sponsor-Investigator 15

1.2 Principal Investigator(s) 15

1.3 Statistician ("Biostatistician") 15

1.4 Laboratory 15

1.5 Monitoring institution 15

1.6 Data Safety Monitoring Committee 16

1.7 Any other relevant Committee, Person, Organisation, Institution 16

2. ETHICAL AND REGULATORY ASPECTS 17

2.1 Study registration 17

2.2 Categorisation of study 17

2.3 Competent Ethics Committee (CEC) 17

2.4 Competent Authorities (CA) 17

2.5 Ethical Conduct of the Study 17

2.6 Declaration of interest 17

2.7 Patient Information and Informed Consent 17

2.8 Participant privacy and confidentiality 18

2.9 Early termination of the study 18

2.10 Protocol amendments 18

3. Background and Rationale 19

3.1 Background and Rationale 19

State of Research 19

Past Work by the Research Team 20

Study 1: Physicians’ Communication Goals 20

Study 2: Patient Endorsement of Physicians’ Communication Goals and Development of the InFARCt Categorization Scheme 20

Study 3: Assessment of Discharge Communication at the University Hospital Basel 24

Study 4: Structured Information and Information Recall 24

Summary of Past Work by the Research Team 25

3.2 Investigational Product (treatment, device) and Indication 27

3.3 Preclinical Evidence 27

3.4 Clinical Evidence to Date 27

3.5 Dose Rationale / Medical Device: Rationale for the intended purpose in study (pre-market MD) 27

3.6 Explanation for choice of comparator (or placebo) 27

3.7 Risks / Benefits 27

3.8 Justification of choice of study population 27

4. STUDY OBJECTIVES 29

4.1 Overall Objective 29

4.2 Primary Objective 29

4.3 Secondary Objectives 29

4.4 Safety Objectives 29

5. STUDY OUTCOMES 29

5.1 Primary Outcome 29

5.2 Secondary Outcomes 29

5.3 Other Outcomes of Interest 29

5.4 Safety Outcomes 29

6. STUDY DESIGN 30

6.1 General study design and justification of design 30

6.2 Methods of minimising bias 30

6.2.1 Randomisation 30

6.2.2 Blinding procedures 30

6.2.3 Other methods of minimising bias 30

6.3 Unblinding Procedures (Code break) 31

7. STUDY POPULATION 32

7.1 Eligibility criteria 32

7.2 Recruitment and screening 33

7.3 Assignment to study groups 33

7.4 Criteria for withdrawal / discontinuation of participants 33

8. STUDY INTERVENTION 34

8.1 Identity of Investigational Products (treatment / medical device) 34

8.1.1 Experimental Intervention (treatment / medical device) 34

8.1.2 Control Intervention (standard/routine/comparator treatment / medical device) 34

8.1.3 Packaging, Labelling and Supply (re-supply) 34

8.1.4 Storage Conditions 34

8.2 Administration of experimental and control interventions 34

8.2.1 Experimental Intervention 34

8.2.2 Control Intervention 34

8.3 Dose / Device modifications 34

8.4 Compliance with study intervention 34

8.5 Data Collection and Follow-up for withdrawn participants 34

8.6 Trial specific preventive measures 34

8.7 Concomitant Interventions (treatments) 34

8.8 Study Drug / Medical Device Accountability 35

8.9 Return or Destruction of Study Drug / Medical Device 35

9. STUDY ASSESSMENTS 35

9.1 Study flow chart(s) / table of study procedures and assessments 35

9.2 Assessments of outcomes 35

9.2.1 Assessment of primary outcome 36

9.2.2 Assessment of secondary outcomes 37

9.2.3 Assessment of other outcomes of interest 37

9.2.4 Assessment of safety outcomes 37

9.2.5 Assessments in participants who prematurely stop the study 37

9.3 Procedures at each visit 37

9.3.1 Pre-discharge assessment 37

9.3.2 Interview 37

9.3.3 Telephone or personal interview 1&2 37

10. SAFETY 38

10.1 Drug studies 38

10.1.1 Definition and assessment of (serious) adverse events and other safety related events 38

10.1.2 Reporting of serious adverse events (SAE) and other safety related events 38

10.1.3 Follow up of (Serious) Adverse Events 38

10.2 Medical Device Category C studies 38

10.2.1 Definition and Assessment of (Serious) Adverse Events and other safety related events 38

10.2.2 Reporting of (Serious) Adverse Events and other safety related events 38

10.2.3 Follow up of (Serious) Adverse Events 38

10.3 Medical Device Category A studies 38

10.3.1 Definition and Assessment of safety related events 38

10.3.2 Reporting of Safety related events 38

11. STATISTICAL METHODS 39

11.1 Hypothesis 39

11.2 Determination of Sample Size 39

11.3 Statistical criteria of termination of trial 39

11.4 Planned Analyses 39

11.4.1 Datasets to be analysed, analysis populations 39

11.4.2 Primary Analysis 39

11.4.3 Secondary Analyses 39

11.4.4 Interim analyses 39

11.4.5 Safety analysis 40

11.4.6 Deviation(s) from the original statistical plan 40

11.5 Handling of missing data and drop-outs 40

12. QUALITY ASSURANCE AND CONTROL 40

12.1 Data handling and record keeping / archiving 40

12.1.1 Case Report Forms 40

12.1.2 Specification of source documents 40

12.1.3 Record keeping / archiving 40

12.2 Data management 41

12.2.1 Data Management System 41

12.2.2 Data security, access and back-up 41

12.2.3 Analysis and archiving 41

12.2.4 Electronic and central data validation 41

12.3 Monitoring 41

12.4 Audits and Inspections 41

12.5 Confidentiality, Data Protection 41

12.6 Storage of biological material and related health data 41

13. PUBLICATION AND DISSEMINATION POLICY 41

14. FUNDING AND SUPPORT 42

14.1 Funding 42

14.2 Other Support 42

15. INSURANCE 42

16. REFERENCES 43

Study synopsis

| **Sponsor/Sponsor-Investigator** | Prof. Dr. Roland Bingisser  University Hospital of Basel  Petersgraben 2  4031 Basel  Switzerland |
| --- | --- |
| **Study Title:** | Improving Discharge Communication in the Emergency Department through Information Structuring: A Cluster Randomized Controlled Trial |
| **Short Title/Study ID:** | Improving Discharge Communication |
| **Protocol Version and Date:** | 02; 02/01/2015 |
| **Trial Registration:** | As soon as the ethics committee tells us to register. |
| **Study Category with Rationale:** | Clinical study, because the participants are patients with chest pain and patients with abdominal pain. The rationale for focusing on these target groups is twofold: First, chest pain and abdominal pain are common complaints, requiring efficient handling to maximize patient care while ensuring efficient use of resources. Second, patients with chest pain and patients with abdominal pain complaints often need additional work-up and follow-up (including treatment), which makes adequate discharge communication crucial. |
| **Clinical Phase:** | NA |
| **Background and Rationale:** | In an emergency department, effective discharge communication—i.e., information on diagnoses and instructions on further management at the time of discharge—represents a key step in medical care, with the potential to improve patient well-being and satisfaction, adherence to medication, and, ultimately, better health outcomes. Past research suggests that physicians’ communication is often not optimally structured and that patients fail to fully recall and understand the information received at discharge. In turn, psychological research suggests that information structuring can be a powerful tool to improve memory recall and understanding. Consequently, information structuring represents a tool to improve discharge communication and associated patient outcomes. |
| **Objective(s):** | The goal of the proposed study is to assess the potential of information structuring for improving discharge communication. Specifically, we aim to examine the advantages of an information-structuring skills training for physicians (compared to an emotion skills training) on discharge communication and associated patient outcomes, such as patients’ information recall and adherence to physician recommendations. We hypothesize that patients receiving structured discharge information from their trained physicians will be able to recall more information and show higher adherence to recommendations relative to controls (i.e., patients receiving discharge information from doctors trained in emotion-handling skills). |
| **Outcome:**  **Primary Outcome**  **Secondary Outcome** | Our primary outcome of interest is patients’ recall of the information provided during discharge consultations. In addition, a number of secondary outcomes associated with patient understanding of and adherence to physicians’ recommendations will be assessed. Recall performance is positively associated with comprehension^26^ and knowledge is a precondition for, albeit not synonymous with,^27^ compliance. Consequently, we hypothesize that recall performance will be positively correlated with patients’ understanding and adherence to physician’s recommendations.  Secondary outcomes:  -higher patient satisfaction  -more reliable medication intake  -more reasonable disease management |
| **Study Design:** | The study will use a cluster randomized controlled trial. Clusters of physicians (cohorts of six physicians commencing training in a three-month period; total of four clusters in the one-year intervention period) will be assigned to one of two types of training (information-structuring skills vs. emotion-handling skills). The primary outcome of the study will be patients’ recall of information as a function of physicians’ training. For this purpose, we aim to recruit 50 patients presenting to the emergency department with chest pain and 50 patients with abdominal pain in each three-month period (N = 400, in the one-year study period) and document the respective discharge communication (via audio recordings). In addition, each patient will be asked to recall the discharge information immediately after the discharge event, seven days and 30 days later, as well as complete a number of other measures to assess secondary outcomes, such as overall satisfaction and adherence to recommendations. The study will be conducted at the emergency department of the University Hospital of Basel (Switzerland). |
| **Inclusion/Exclusion Criteria:** | We will train a total of 24 physicians. The intervention will be assessed by interviewing a total of 400 patients who present to the emergency department of the University Hospital Basel with chest pain or with abdominal pain and who are seen by one of the study participants.  We will use the following exclusion criteria concerning patients with chest pain:   - Patients *younger than 18 years of age* will not be recruited because of limited ability to provide informed consent. - Patients with *limited ability to communicate in German* (the default language at the hospital) will not be recruited to exclude additional confounds related to language proficiency. - Patients with *dementia* will not be recruited to avoid additional confounds arising from pathological memory deficits. - Patients with *increased troponin level*, an indicator of myocardial infarction, will not be recruited to minimize stress on these more vulnerable patients. - Patients with none of five *cardiovascular risk factors* (cvRF; smoking history, diabetes, hypertension, dyslipidemia, age above 50 years, or family history of coronary heart disease) will not be recruited because chest pain symptoms in such patients are typically indicative of diagnoses unrelated to coronary heart disease, requiring a very different follow-up and health management recommendations (e.g., in the case of an anxiety disorder).   We will use the following exclusion criteria concerning patients with abdominal pain:   - Patients *younger than 18 years of age* will not be recruited because of limited ability to provide informed consent. - Patients with *limited ability to communicate in German* (the default language at the hospital) will not be recruited to exclude additional confounds related to language proficiency. - Patients with *dementia* will not be recruited to avoid additional confounds arising from pathological memory deficits. |
| **Measurements and Procedures:** | Study Procedure  The study involves two components: (a) the intervention component, consisting of the two physician-training programs and (b) the evaluation component, consisting of an assessment of the interventions through patient testing and interviews.  Physician Training  Physicians will be recruited to participate in the study, which will serve as a complement to physicians’ regular emergency department training. The two programs will follow the same structure, with physicians receiving the same intensity of teaching. Both programs will consist of one four-hour session of training with an experienced communication expert (Prof Dr Wolf Langewitz). Also, both training programs will consist of theory and practice components as well as a test session to assess the actual use of communication skills. The information-structuring program will include a review of scientific evidence on the superiority of structured over non-structured communication as well as hands-on practical exercises in dyads to train structured communication skills. Specifically, participants will be introduced to the ‘InFARcT-scheme’ (categorization scheme that could, in principle, help physicians to structure information; for more information see: past work by research team) and trained to use it to structure their communication. The emotion skills training program will follow a similar structure but will focus on the NURSE communication model.^28^ The NURSE model encourages caretakers to engage in naming, understanding, respecting, supporting, and exploring patients’ emotional reactions with the goal of reducing anxiety and increasing compliance. In addition, physicians in both conditions will receive a one-hour booster session two weeks after the initial training involving a discussion of patient examples to whom they had applied the newly acquired skills. The rationale for the use of a booster session is to ensure that communication goals are maintained as a high priority and that physicians have a chance to receive additional feedback early in their training.  Patient Interviews  All physicians participating in the study will be asked to contact a member of the study team about any pending discharge communication with a patient suffering from chest pain or a patient suffering from abdominal pain. Eligible participants (see section on Participants) will be asked to give written consent to participate in the study and complete a pre-discharge assessment. The pre-discharge assessment will take about 15 minutes and aims to collect basic demographic measures concerning each patient as well as a number of other measures that will serve as covariates in the analysis, such as patients’ health status, memory ability, state anxiety, state depression, and medical knowledge. The rationale for including these measures is that they are potential moderators of participants’ recall of discharge information and thus should be included in the statistical analysis.  Immediately after the discharge communication event, patients will be interviewed for up to five minutes to assess recall of information provided during the discharge consultation and to assess patients’ satisfaction with the encounter.  Finally, two brief interviews of 5–10 minutes will be conducted over the phone seven days after discharge and 30 days after discharge to retest recall of discharge information, thus assessing the reliability of the main outcome measure and the time component of recall. In addition, patients will be asked about self-reported adherence to discharge recommendations, and overall assessment of the discharge consultation. Table 4 provides a description of the measures collected in each phase. Patients will also be asked about information provided by their General Practitioners to control for possible confounds of receiving extra or additional information from a third-party.  The discharge communication encounter as well as all interviews will be recorded with audio recording devices. |
| **Study Product/Intervention according to KlinV, if applicable:** | No medicaments are additionally being given to the patients. The intervention is the communication skills training for the physicians. |
| **Comparator(s) (if applicable):** | We will compare the effects of information structuring to a control training condition in which physicians will receive emotion skills training by the same experts. A systematic review of emotion skills training suggests that these have the potential to improve physician–patient interactions.^23^ However, emotion skills interventions are not geared to changing the structure of the information provided and thus represent a powerful control for our study. In particular, it will ensure that any effects in information recall and patient satisfaction can be attributed to the information structuring program rather than a general emphasis on communication training and patient satisfaction during physician training. |
| **Number of Participants with Rationale (if no Power Analysis conducted):** | Within three months 50 patients with chest pain and 50 patients with abdominal pain will be recruited. Including 4 groups with 100 patients each this sums up in 400 patients in one year. Sample of 400 gives acceptable power of .8 for comparison of two groups.  We based our sample sizes on calculations based on previous studies that compared an information structuring intervention relative to treatment as usual and suggest a medium effect size d = .59 for the intervention. To compute power for our planned analysis we conducted simulations in which we generated data using the means and standard deviations as those observed in the previous study but let patients be sampled from an homogenous pool of 6 physicians and varied the sample sizes per intervention group from 40 to 120. We then conducted 1000 repeated iterations for each sample size and analysed the data with a mixed effects model of patient’s recall clustered by physician and calculated the frequency of observing a significant effect of intervention for each sample size. A sample size of 100 participants per condition guarantees an appropriate power of over .9. Please note that while a smaller sample sizes per condition of over 50 would guarantee satisfactory power levels above .8, having a larger initial sample size is of importance given the possible attrition rates between the first measurement point (immediately after discharge) and the two planned follow-ups (over the telephone). Specifically, our desired sample size guarantees that we would have power above .9 for the analysis of the follow-up measurements in the face of 25% attrition rates over measurement occasions. |
| **Study Duration:** | The study will start at 02/01/2015 and will end at 31/12/2015 (=1 year). |
| **Study Schedule:** | First contact with study participants will be the 02/01/2015 when Prof. Dr. Wolf Langewitz will teach the first cohort of physicians. The last contact with participants will be the last doctor patient communication (approx. 31/12/2015). |
| **Investigator(s):** | Prof. Dr. Roland Bingisser  Universitätsspital Basel  Interdisziplinäre Notfallstation  Petersgraben 2  4031 Basel  Schweiz  PD Dr. Christian Nickel  Universitätsspital Basel  Petersgraben 2  4031 Basel  Schweiz  Victoria Siegrist  Interdisziplinäre Notfallstation  Petersgraben 2  4031 Basel  Schweiz |
| **Study Centre(s):** | Emergency Department  University Hospital of Basel  Petersgraben 2  4031 Basel  Switzerland |
| **Statistical Analysis incl. Power Analysis** | The evaluation of the effects of the two training programs will be conducted using mixed-effects model.^33^ Mixed-effects models are statistical models containing both fixed (e.g., intervention program) and random (e.g., patient) effects and are used in a variety of disciplines including medical and social sciences, where repeated measurements are made on the same statistical units or on clusters of related statistical units. Specifically, our analysis will take into account the fact that physicians belong to particular clusters (see Study Design) and the same physician treats several patients, by clustering physicians by training program and patients by physician. These multivariate analysis methods also allow for simultaneous statistical adjustment for covariates of interest, including patient characteristics, such as age, memory ability, and so on. Similar models will be used to estimate the effect of the training programs on patient satisfaction, and adherence to discharge recommendations. |
| **GCP Statement:** | This study will be conducted in compliance with the protocol, the current version of the Declaration of Helsinki, the ICH-GCP or ISO EN 14155 (as far as applicable) as well as all national legal and regulatory requirements. |

study summary in local language

Allgemeine Informationen:

Wir führen eine Studie mit vierhundert Patienten durch, die zum Ziel hat, die Kommunikation zwischen Ärzten und Patienten, die mit Thoraxschmerzen oder Bauchschmerzen zu uns auf die Notfallstation kommen, zu verbessern.

Ziel der Studie:

Adäquate Informationsvermittlung beim Austritt eines Patienten nach einem Krankenhausaufenthalt kann einen grossen Einfluss auf die Lebensqualität des Patienten sowie auf andere Aspekte wie die Rehospitalisierungrate und die Gesundheitskosten haben. Die bisherige Forschung konzentrierte sich vor allem auf die Form/Art der Austritts-Informationsvermittlung (z.B. mündlich vs. schriftlich), der Darbietung solcher Informationen, insbesondere im Notfall-Setting, wurde bis anhin kaum erforscht. Unser Ziel ist es deshalb, die Darbietung der Austrittskommunikation (bei Patienten mit Thoraxschmerzen und bei Patienten mit Bauchschmerzen) zu ermitteln, welche, aus gesundheitspolitischer Sicht, zu verbesserten Outcomes führt.

Study schedule

We plan to conduct a cluster randomized controlled trial to test the effect of two physician training programs on patient information recall, namely, an information structuring training and an emotion skills training. Physicians that receive training at the University Hospital Basel usually stay for a six-month period in the emergency department before rotating to another department, with a new clusters of six physicians initiating training every three months. Consequently, the study will run for one year and involve four different clusters of physicians to ensure that sufficient physicians and associated patients can be included in the study (see also section on Participants below). Clusters of physicians will thus be pseudo-randomly assigned to one of the two training programs (information structuring vs. emotion-handling skills training). Patients attended by physicians from each respective cluster will be tested and interviewed to provide an assessment of the effects of the two training programs.

There will be a pre-discharge assessment, an interview immediately after the discharge event and a telephone interview one week and 30 days after the discharge event.

Training/Interviewing Timeline for Each Cluster of Physicians (*k*)/Patients (*n*)

| Cluster | Months 1-3 | Months 4-6 | Months 7-9 | Months 10-12 |
| --- | --- | --- | --- | --- |
| 1 | Information Structuring  (*k*=6, *n* = 100) |  |  |  |
| 2 |  | Emotion Skills  (*k*=6, *n* = 100) |  |  |
| 3 |  |  | Information Structuring  (*k*=6, *n* = 100) |  |
| 4 |  |  |  | Emotion Skills  (*k*=6, *n* = 100) |

# STUDY ADMINISTRATIVE STRUCTURE

## Sponsor, Sponsor-Investigator

Prof. Dr. Bingisser is the principal investigator (PI) and this is an investigator-initiated study.

Prof. Dr. Roland Bingisser

Universitätsspital Basel

Interdisziplinäre Notfallstation

Petersgraben 2

4031 Basel

Schweiz

Phone: +41 61 265 58 30

## Principal Investigator(s)

Prof. Dr. Roland Bingisser

Universitätsspital Basel

Interdisziplinäre Notfallstation

Petersgraben 2

4031 Basel

Schweiz

Phone: +41 61 265 58 30

## Statistician ("Biostatistician")

Clinical Trial Unit

Thomas Fabbro

University Hospital Basel

Schanzenstrasse 55

4031 Basel

Switzerland

Phone: +41 61 328 77 74

## Laboratory

NA, this is a clinical study.

## Monitoring institution

Clinical Trial Unit

Thomas Fabbro

University Hospital Basel

Schanzenstrasse 55

4031 Basel

Switzerland

Phone: +41 61 328 77 74

## Data Safety Monitoring Committee

No interim analysis is planned. No safety monitoring board has been called.

## Any other relevant Committee, Person, Organisation, Institution

University of Basel

Faculty of Psychology

Department of Cognitive and Decision Sciences

Prof. Dr. Rui Mata

Missionsstrasse 64A

4055 Basel

Switzerland

Phone: +41 61 267 06 11

# ETHICAL AND REGULATORY ASPECTS

## Study registration

AS soon as the ethics committee tells us to register, we’ll register at https://register.clinicaltrials.gov.

## Categorisation of study

The risk category is A. The rationale for this categorisation is the study design. We don’t use additional medication, we just improve the physician’s training to get better outcomes on the physician’s patient communication.

## Competent Ethics Committee (CEC)

The principal investigator ensures the approval from an appropriately constituted Competent Ethics Committee (CEC) is sought for the clinical study

Premature study end or interruption of the study will be reported within 15 days. The regular end of the study will be reported to the CEC within 90 days, the final study report will be submitted within one year after study end. Amendments will be reported according to chapter 3.10.

## Competent Authorities (CA)

NA, Category A study.

## Ethical Conduct of the Study

The study will be carried out in accordance to the protocol and with principles enunciated in the current version of the Declaration of Helsinki, the guidelines of Good Clinical Practice (GCP) issued by ICH, in case of medical device: the European Directive on medical devices 93/42/EEC and the ISO Norm 14155 and ISO 14971, the Swiss Law and Swiss regulatory authority’s requirements. The CEC and regulatory authorities will receive annual safety and interim reports and be informed about study stop/end in agreement with local requirements.

## Declaration of interest

There is no conflict of interest.

## Patient Information and Informed Consent

The investigators will explain to each participant the nature of the study, its purpose, the procedures involved, the expected duration, the potential risks and benefits and any discomfort it may entail. Each participant will be informed that the participation in the study is voluntary and that he/she may withdraw from the study at any time and that withdrawal of consent will not affect his/her subsequent medical assistance and treatment.

The participant will be informed that his/her medical records may be examined by authorised individuals other than their treating physician.

All participants for the study will be provided a participant information sheet and a consent form describing the study and providing sufficient information for participant to make an informed decision about their participation in the study. Enough time will be given to the participant to decide whether to participate or not. After reading the information sheet and the consent form there will be time for asking questions and the possibility to think about the decision for some minutes.

The patient information sheet and the consent form will be submitted to the CEC to be reviewed and approved. The formal consent of a participant, using the approved consent form, will be obtained before the participant is submitted to any study procedure.

The participant should read and consider the statement before signing and dating the informed consent form, and will be given a copy of the signed document. The consent form must also be signed and dated by the investigator and it will be retained as part of the study records.

## Participant privacy and confidentiality

The investigator affirms and upholds the principle of the participant's right to privacy and that they shall comply with applicable privacy laws. Especially, anonymity of the participants shall be guaranteed when presenting the data at scientific meetings or publishing them in scientific journals.

Individual subject medical information obtained as a result of this study is considered confidential and disclosure to third parties is prohibited. Subject confidentiality will be further ensured by utilising subject identification code numbers to correspond to treatment data in the computer files.

For data verification purposes, authorised representatives of the Sponsor (-Investigator), a competent authority (e.g. Swissmedic), or an ethics committee can require direct access to parts of the medical records relevant to the study, including participants’ medical history.

## Early termination of the study

The Sponsor-Investigator may terminate the study prematurely according to certain circumstances:

- ethical concerns,
- when the safety of the participants is doubtful or at risk, respectively,
- alterations in accepted clinical practice that make the continuation of a clinical trial unwise,
- early evidence of benefit or harm of the experimental intervention

## Protocol amendments

Substantial amendments are only implemented after approval of the CEC and CA respectively.

Under emergency circumstances, deviations from the protocol to protect the rights, safety and well being of human subjects may proceed without prior approval of the sponsor and the CEC/CA. Such deviations shall be documented and reported to the sponsor and the CEC/CA as soon as possible.

All Non-substantial amendments are communicated to the CA as soon as possible if applicable and to the CEC within the Annual Safety Report (ASR).

# Background and Rationale

## Background and Rationale

## State of Research

Discharge from the emergency department is a period of high vulnerability for many patients.^1^ Inappropriate communication at discharge may result in adverse events, most often related to incorrect adherence to medication, and a lack of follow-up related to pending test results, highlighting the central role of emergency physicians facilitating continuity of care and as a key link to the primary care provider.^2,3^ Effective physician–patient communication at discharge, by which patients can recall and understand medical information is therefore a crucial part of patient care leading to improved patient outcomes, including higher patient satisfaction,^4^ better adherence to medication,^4,5^ more adequate disease management,^6^ and reduced anxiety.^7^ Unfortunately, effective discharge communication appears to be the exception rather than the rule: Even in immune-compromised patients, for which knowledge of medication is crucial to treatment success, knowledge of recommendations at discharge is merely moderate.^4^

Chest pain accounts for up to 10% of all patient encounters in emergency departments.^8^ However, many patients with chest pain presenting to an emergency department are discharged within hours, once serious conditions, such as myocardial infarction, have been excluded and when further evaluation of low-to-intermediate risk patients is not possible in the context of the emergency department.^9^ Additionally, after the emergency department work-up and discharge, many patients have repeated episodes of chest pain, often associated with anxiety and uncertainty about diagnosis and outcome.^10^ Patients complaining of chest pain may suffer from coronary heart disease, and, for these patients, the discharge process represents an important transition to primary care with adherence to recommendations playing a crucial role in adequate treatment.^3^ A recent study assessing the experiences of chest pain patients after discharge revealed that patients often did not recall receiving a definitive diagnosis or advice and reported limited opportunities to discuss the diagnosis, their particular worries, and further management.^11^ A study using telephone interviews to assess the recall of discharge instructions in patients with acute coronary syndrome demonstrated that many patients were unable to name their diagnosis or understand the link between personal risk factors and other contributing causes.^12^

Systematic literature reviews suggest that there are a number of possible interventions that may improve discharge communication.^1,13^ Samuels-Kalow et al. (2012) suggest that communication should (1) be standardized, (2) be adapted to the patient’s knowledge and language, (3) include comprehension checks, and (4) involve patient reminders or help with follow-up appointments, among others.^1^ One possible way to standardize communication is to provide written information^14^; however, this is not always possible when information needs to be tailored to a specific patient, patient literacy is low, or diagnoses are varied or unclear—as is often the case with chest pain complaints. A more feasible alternative is, therefore, to ensure that the typical oral communication includes the main characteristics listed above.

Psychological theory and associated empirical findings suggest that information structuring can be a powerful tool in improving memory recall and understanding. In a seminal study investigating the influence of structure on learning, Epstein (1967) showed that verbal structured material was learned and later recalled better than unstructured material.^15^ Meta-analyses on the use of a specific type of information structuring, namely, advance organizers (i.e., information presented by an instructor prior to learning with the goal of helping the learner organize new incoming information), suggest that structure can indeed assist learning: Hattie (2009) estimated an overall positive effect size of .4 on learning from 11 meta-analyses of 577 studies (*N* = 3905).^16^ One likely psychological mechanism underlying the benefits of information structuring appears to be chunking; that is, the grouping of disparate individual low-level elements into high-level clusters.^17^ Indeed, the ability to form high-level clusters has been directly linked to increases in recall capacity, making it a useful tool for memorizing large amounts of information.^18-20^

The power of information structuring and associated chunking mechanisms has been mostly studied in a laboratory environment and, to date, no studies have investigated its use in improving discharge information delivery. Could information structuring also improve patients’ recall and understanding of discharge information, and, ultimately, patients’ adherence to recommendations? If so, how should doctors best structure information at discharge to achieve these goals? How do possible effects on patients’ recall translate into better adherence to recommendations?

## Past Work by the Research Team

We have conducted a number of studies laying the groundwork for assessing the potential of information structuring in improving discharge communication. Specifically, we conducted studies focusing on the content of discharge information as perceived by physicians (Study 1), the relevance of these goals to patients’ information needs (Study 2), an assessment of current discharge communication practices in the emergency department of the University Hospital Basel (Study 3), and the effects of information structuring on lay and expert populations (psychology and medical students; Study 4). Overall, the results of these four studies suggest that physicians’ communication goals are ambitious but shared with patients, that current discharge communication is not optimal, and that information structuring is a plausible intervention with the potential to improve recall of information at discharge. We present each of the studies and our conclusions below.

### Study 1: Physicians’ Communication Goals

The goal of **Study 1** was to document physicians’ communication goals, specifically, the number of items that physicians deemed important to cover in a typical discharge communication and time needed for this procedure.^21^ We first asked seven expert physicians with over ten years’ experience to list all possible pieces of information to be disclosed to patients in a discharge communication event (given infinite time). This yielded a total of 81 items. In the next step, we presented a case vignette describing a 63-year-old patient with chest pain to 47 physicians with a background in emergency medicine and internal medicine. We then asked participants to choose those items from the 81-item list that they thought were crucial to be given to the patient within a 15-minute discharge consultation. Physicians chose, on average, 36 of the 81 items (SD = 9.8; range: 20–57). We also asked experts to rate the time necessary to communicate these 36 items and obtained an estimate of about 45 minutes—almost three times the preset 15-minute time limit. Overall, these results show that most physicians have problems adapting the number of information items to the time constraints of an emergency department: Physicians in our study proved to be poorly calibrated with regard to the number of items they could realistically discuss in a 15-minute discharge communication event. These results suggest that there is a need to train physicians concerning efficient discharge communication.

### Study 2: Patient Endorsement of Physicians’ Communication Goals and Development of the InFARCt Categorization Scheme

**Study 2** tested whether patients endorsed the list of items that physicians had chosen to be communicated at discharge. We also aimed to develop a categorization scheme that could, in principle, help physicians to structure information.

Shortly before the physician started the discharge communication, we conducted interviews with 51 patients due to be discharged from the emergency department after having presented with complaints of chest pain,. Patients were presented with a list of 34 potential items and asked to select those they deemed most important. This list was obtained by selecting the items from Study 1 (N=81) that were chosen by more than 50% of physicians. Table 1 presents all items and the percent of physicians and patients that endorsed each item. As can be seen in Table 1, all but 2 of the 34 items endorsed by the majority of physicians were also judged to be important by the majority of patients, suggesting that patients are motivated and interested in acquiring the information provided by physicians at discharge.

Our second aim was to synthesize the information into the smallest number of discrete categories that capture all elements that the physicians and patients thought were important. Given the high concordance between physicians and patients, we used the condensed list of 34 items to generate categories. Three expert physicians, with experience of more than 12 years in the field of emergency medicine, as well as teaching and training of junior physicians, aimed to identify a small number of non-overlapping basic categories to which the individual items could be assigned. The three experts worked individually to classify each item. Each individual classification system was then shared and discussed with the others with the goal of arriving at a system agreed upon by all three experts. The resulting classification system comprises five categories (see Table 1), namely, “Information on diagnosis,” “Follow-up suggestions”, “Advice on self-care”, “Red flags”, and “Complete treatment.”

We also created a mnemonic device, the InFARcT acronym, using the initial letter(s) from the classification categories. This acronym is not a neologism but represents a word with an established meaning and highly pertinent to patients with acute chest pain. There is a tradition of using such mnemonics to help physicians and other health practitioners recall particular cues or procedures, albeit their effectiveness is often not systematically tested.^22^ We hoped this mnemonic device could be deployed to help physicians structure their discharge communication events with patients with chest pain complaints. In the next studies we conducted the first steps to establish its need in a clinical setting (Study 3) and possible effects on memory recall (Study 4).


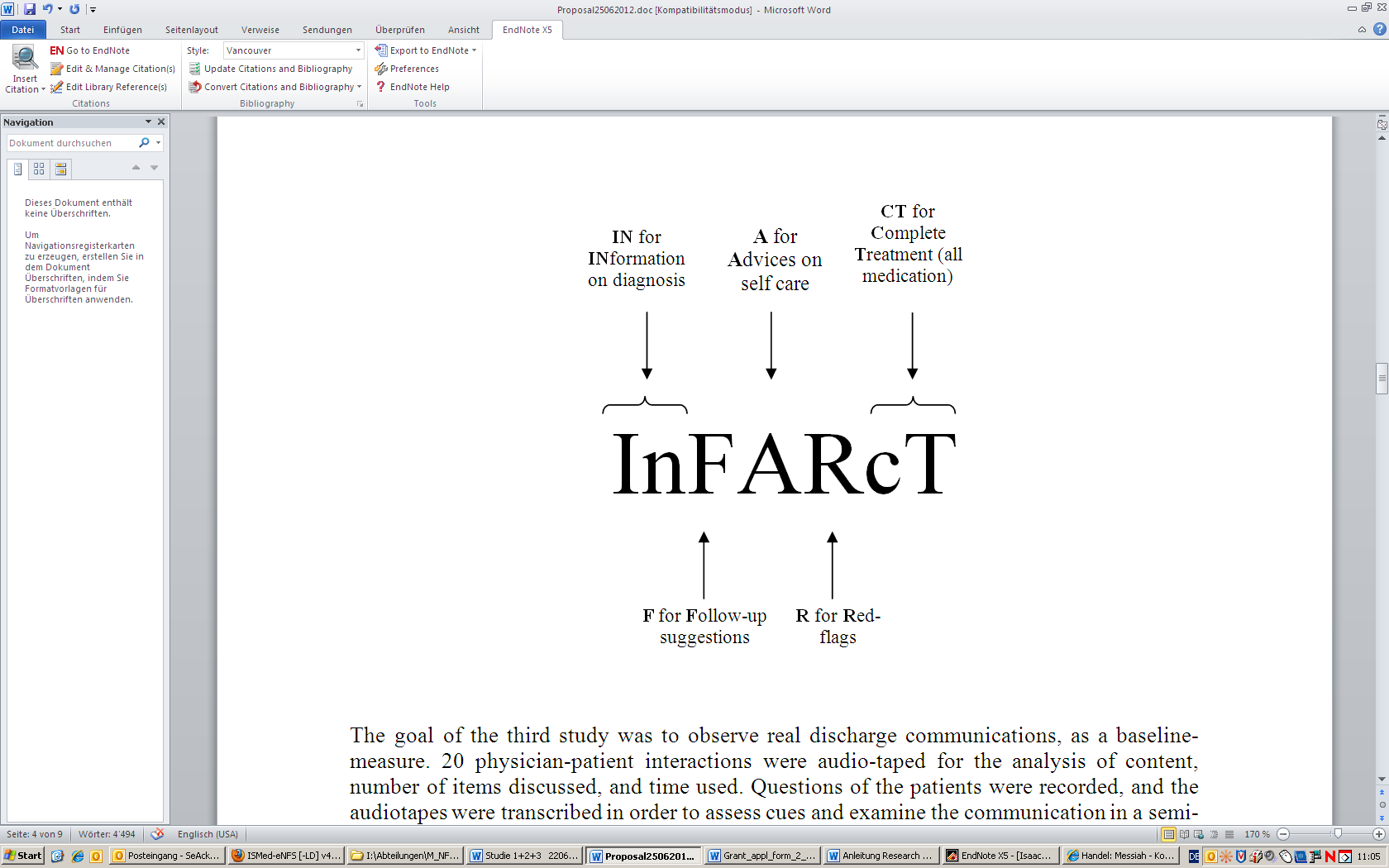


*Figure 1. Mnemonic Device: The InFARcT Acronym*

Table 1. InFARCt Categories, respective 34 Items, and Item Endorsement by Physicians (Study 1) and Patients (Study 2).

| Category | Item | Percent Endorsed | |
| --- | --- | --- | --- |
|  |  | Physicians (N = 47) | Patients (N = 51) |
| Information on diagnosis  (7 items) | Inform the patient that he is ready to go home  Reassure the patient (“you were right to come to the Emergency Department”)  Explaining that blood, heart, and lungs were thoroughly examined  State the presumptive diagnosis  Broad statement: “All the investigations exclude a diagnosis of myocardial infarction at this time”  Explain the significance of the presumptive diagnosis  Explain the association of symptoms with the suspected diagnosis | 89  72  57  83  79  66  62 | 96  73  100  98  94  96  96 |
| Follow-up suggestions  (9 items) | State why further investigation is necessary  State what the planned investigations are  State when the investigations will be carried out  State where the investigations will be done  Describe necessary precautions for the test (no coffee, no tea)  Explain that an information sheet with details of the pre-test preparation will be sent by post  Explain that detailed information on the time and location of the test will be sent by post  Advise the patient to contact his family physician should he have further questions  Encourage the patient to make an appointment with his family physician to obtain more information | 94  89  77  74  64  57  68  79  68 | 92  75  88  82  88  65  78  65  50 |
| Advice on self-care  (4 items) | Address risk factors  Address the need to stop smoking  Address current avoidance of physical stress  Recommend that the patient resumes normal daily activities | 53  83  81  53 | 94  48  78  90 |
| Red flags  (6 items) | Stress that the patient should present immediately to the ED in case of chest pain radiating into arms/jaws  Stress that the patient should present immediately to the ED if the symptoms last longer than 10 minutes  Stress that the patient should present immediately to the ED if he is dyspnoeic  Stress that the patient should present immediately to the ED if he experiences chest pain not responding to nitroglycerine  Explain that the ED is open 24/7 (“you may come back any time”)  Reassert the importance of presenting immediately to the ED in case of any complaints or symptoms, even at night | 83  81  68  96  68  57 | 94  86  92  88  63  53 |
| Complete treatment  (medication; 8 items) | Explain that treatment has to start immediately  Explain why treatment has to start immediately  State the names of the new medications (ASS, beta blocker, nitroglycerine spray)  Give the ASS dose and explain when it should be taken  Give the beta blocker dose and explain when it should be taken  Describe the side effects of beta blockers  Give the nitroglycerine dose and explain when it should be taken  Describe the side effects of nitroglycerine | 55  70  96  66  64  53  81  62 | 90  86  76  84  88  85  86  89 |

### Study 3: Assessment of Discharge Communication at the University Hospital Basel

The goal of **Study 3** was to describe the content and quality of the current discharge communication at the University Hospital Basel. For this purpose, we audio-recorded 20 examples of discharge communication involving patients with chest pain. Communication was transcribed and coded by two judges for presence of the InFARCt-categories and for the quality of communication. Additionally, raters determined whether physicians offered the patient an opportunity to ask questions and whether the patient’s comprehension was checked. The results showed that discharge communications took on average six minutes. As depicted in Table 2, all patients were given information on their diagnosis and follow-up (100%) and most patients were given information on how to complete treatment (75%). However, only half of the patients (50%) were given instructions on “red-flags” and only a minority received advice on self-care (36%). Importantly, the quality of the communication was often only adequate or minimal and there was often no opportunity to ask questions; checking patients’ comprehension was quite uncommon. Overall, these results suggest there is considerable heterogeneity in the quality and content of information provided at discharge and that interventions to improve communication are desirable.

Table 2. Percent and Quality of Information Types Provided During Discharge Communication

| Category | Included | Minimal | Adequate | Excellent |
| --- | --- | --- | --- | --- |
| Information on diagnosis | 100 | 15 | 30 | 55 |
| Follow-up suggestions | 100 | 35 | 35 | 30 |
| Advice on self-care | 35 | 15 | 15 | 5 |
| Red-flags | 50 | 20 | 15 | 15 |
| Complete treatment | 75 | 35 | 20 | 20 |
| Opportunity to ask questions | 90 | 35 | 45 | 10 |
| Comprehension check | 20 | 10 | 5 | 5 |

### Study 4: Structured Information and Information Recall

The goal of **Study 4** was to conduct an initial test of the potential of information structuring in improving patients’ recall. In a first step, two videos were produced that demonstrate two types of discharge communication: in one video a physician offers information in a structured way, in another video the same information is given in an unstructured way. Depending on randomization, one of the two videos was then shown to participants and recall was assessed immediately afterwards.. The study included three different groups of participants differing in their medical expertise. Specifically, we recruited first-year psychology and medical students, as well as third-year medical students. The rationale for testing groups that differed in medical expertise was the assumption that structure should benefit mostly those individuals who cannot make use of previous knowledge to build memory chunks.

The study used a 2 (structure: structured vs. unstructured) x 3 (expertise: first-year psychology, first-year medical, third-year medical) between-subjects design. The study was conducted during regular weekly lectures in different auditoriums and written informed consent was obtained from all participants (*N* = 234). The study procedure consisted of giving participants brief written information on the study and requesting them to take the perspective of the patient during the screening of a movie of a physician–patient discharge consultation. As outlined above, participants viewed a video showing an experienced physician presenting content either in a structured fashion (that is, in which information was clustered according to the InFARCt-scheme—the InFARCt video) or, alternatively, in an unstructured fashion (the unstructured video). In both videos, the information consisted of those 28 items from Study 2 that had received the highest ranking. Prior to viewing the videos, participants were not informed that their recall would be assessed later. After viewing the video, they were given five minutes to note all facts that they recalled on a blank piece of paper. Subsequently, participants were asked to rate the physician’s comprehensibleness on a scale from 0 to 10, the dialogue structure, and the participants’ willingness to recommend the physician to family and friends, among other questions.

Our analyses had two aims. First, our main goal was to test the effects of information structuring on information recall and possible interactions with prior expertise. Second, we tested for superiority of information structuring relative to unstructured communication on perceived comprehensibleness and satisfaction. In total, participants recalled 9 of 28 items presented by the emergency physician (range: 0-23 items). An analysis of variance with condition (structured vs. unstructured), group (third-year medical, first-year medical, first-year psychology), and their interaction as factors, revealed a main effect of structure, *F*(1,228)=26.6, *p* < .001, as well as a expertise, F(2,228)=7.0, *p* = .009. As can be seen in Figure 2B, those participants that viewed the video structured according to the InFARCt categories listed 1 to 3 additional pieces of information relative to those who viewed the unstructured video. Also, third-year medical students did considerably better than first-year medical students, who, in turn, outperformed psychology students. Although the condition by expertise interaction was not significant, *F*(2,228)=0.8, *p* = .44, the magnitude of the effects of information structuring seem to vary systematically by expertise: Although only a negligible difference of structured information could be observed in the expert group (13.5 [SD = 4.5] vs. 13.0 [SD = 4.6], Cohen’s *d* = .11), we found a small effect size of information structuring in first-year medical students (9.9 [SD = 5.1] vs. 8.8 [SD = 4.5], Cohen’s *d* = .23), and a medium effect size in first-year psychology students (8.1 [SD = 4.3] vs. 5.7 [SD = 3.7], Cohen’s *d* = .60). In addition, as can be seen in Figure 2C, participants that viewed the InFARCt video provided higher ratings on items assessing comprehensibleness, structure, and willingness to recommend the physician to family and friends.

Overall, these results suggest that participants without expert medical knowledge (i.e., psychology students) can profit from information structuring during discharge communication events. The results are encouraging in suggesting that an intervention aimed at introducing information structuring in physicians’ communication can lead to improved recall of discharge information, perceived understanding, and increased satisfaction with the communication event.

### Summary of Past Work by the Research Team

Our research team has conducted four studies that lay the foundation for the intervention described below. In our past work, we found that physicians have overly ambitious communication goals, planning to discuss over 30 pieces of information during a discharge consultation (Study 1), and that these high goals are shared by patients (Study 2). However, in real interactions, time spent with discharge communication is much more limited than the time allotted in Study 2, and the content and quality of information provision does not match the high goals of experts and patients (Study 3). Finally, experimental work using simulated discharge communication situations suggests that information structuring can be an effective tool to improve patients’ recall of information (Study 4). All in all, these results suggest that there is need for improving discharge communication and that information structuring may be a tool to both standardize communication and improve patients’ recall of information and, consequently, adherence to health recommendations. In what follows, we present our plan to test an intervention that provides training on information structuring to physicians and assesses the associated outcomes in patients’ information recall, satisfaction, and adherence to recommendations.

|  | |
| --- | --- |
| A | B |
| 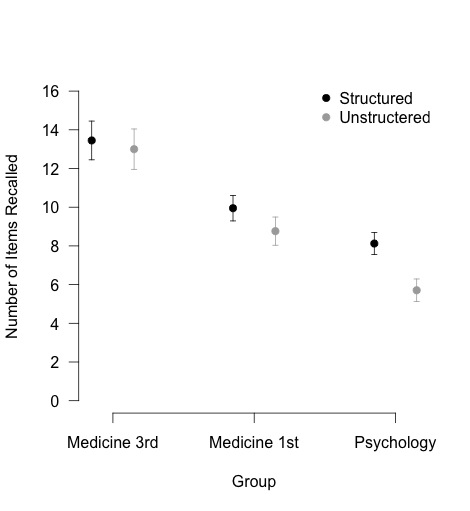 |  |
|  | |

Figure 2. **A** Number of items recalled by participants that viewed either the structured or unstructured video by group (third-year medical, first-year medical, first-year psychology students). **B** Participants’ ratings of physician’s comprehensibleness, information structuring, and willingness to recommend the physician to family and friends as a function of whether they viewed the structured, InFARCt-based video (black icons) or the unstructured video (grey icons).

## Investigational Product (treatment, device) and Indication

**Physician Training**

Physicians will be recruited to participate in the study, which will serve as a complement to physicians’ regular emergency department training. The two programs will follow the same structure, with physicians receiving the same intensity of teaching. Both programs will consist of one four-hour session of training with an experienced communication expert (Prof Dr Wolf Langewitz). Also, both training programs will consist of theory and practice components as well as a test session to assess the actual use of communication skills. The information-structuring program will include a review of scientific evidence on the superiority of structured over non-structured communication as well as hands-on practical exercises in dyads to train structured communication skills. Specifically, participants will be introduced to the InFARcT-scheme and trained to use it to structure their communication. The emotion skills training program will follow a similar structure but will focus on the NURSE communication model.^28^ The NURSE model encourages caretakers to engage in **n**aming, **u**nderstanding, **r**especting, **s**upporting, and **e**xploring patients’ emotional reactions with the goal of reducing anxiety and increasing compliance. In addition, physicians in both conditions will receive a one-hour booster session two weeks after the initial training involving a discussion of patient examples to whom they had applied the newly acquired skills. The rationale for the use of a booster session is to ensure that communication goals are maintained as a high priority and that physicians have a chance to receive additional feedback early in their training.

## Preclinical Evidence

See 3.1

## Clinical Evidence to Date

See 3.1

## Dose Rationale / Medical Device: Rationale for the intended purpose in study (pre-market MD)

NA

## Explanation for choice of comparator (or placebo)

We will compare the effects of information structuring to a control training condition in which physicians will receive emotion skills training by the same experts. A systematic review of emotion skills training suggests that these have the potential to improve physician–patient interactions.^23^ However, emotion skills interventions are not geared to changing the structure of the information provided and thus represent a powerful control for our study. In particular, it will ensure that any effects in information recall and patient satisfaction can be attributed to the information structuring program rather than a general emphasis on communication training and patient satisfaction during physician training.

## Risks / Benefits

We consider the risk of the study being low. There won’t be a direct benefit for the participants considering monetary compensation but previous studies found an effect of physician’s training in students. Therefore there might be a chance, that patients will benefit from physician’s training in both groups (information structuring & emotion skills training) in this intervention study as well..

## Justification of choice of study population

Chest pain accounts for up to 10% of all patient encounters in emergency departments.^8^ However, many patients with chest pain presenting to an emergency department are discharged within hours, once serious conditions, such as myocardial infarction, have been excluded and when further evaluation of low-to-intermediate risk patients is not possible in the context of the emergency department.^9^ Additionally, after the emergency department work-up and discharge, many patients have repeated episodes of chest pain, often associated with anxiety and uncertainty about diagnosis and outcome.^10^ Therefore the reduction of anxiety and uncertainty about the diagnosis is an important issue for this patients. We included patients with abdominal pain because the same applies to patients with abdominal pain.

Signs and symptoms showing that the participant is unwilling to participate in the study will result in the participant being excluded from participation. We guarantee that a physician not participating in the study, safeguards participant interest and insures proper medical care.

# STUDY OBJECTIVES

## Overall Objective

The goal of the proposed study is to assess the potential of information structuring for improving discharge communication. Specifically, we aim to examine the advantages of an information-structuring skills training for physicians (compared to an emotion skills training) on discharge communication and associated patient outcomes.

## Primary Objective

The primary outcome of the study will be patients’ recall of information as a function of physicians’ training.

## Secondary Objectives

The secondary outcomes will be higher patient satisfaction,^4^ better adherence to medication,^4,5^ more adequate disease management,^6^ and reduced anxiety.

## Safety Objectives

The study aims to improve discharge communication and associated patient outcomes.

# STUDY OUTCOMES

## Primary Outcome

Participants’ recall of discharge information will be assessed after the discharge communication and 1 week later. We measure this primary outcome to check whether the intervention was successful or not. We measure the recall 1 week and 30 days later to check the long-term effect of the intervention.

## Secondary Outcomes

Satisfaction with the discharge communication event and the physician directly after the discharge event. The rationale for including this measure is that it is a potential moderator of participants’ recall of discharge information and thus should be included in the statistical analysis.

## Other Outcomes of Interest

Basic demographic measures concerning each patient as well as a number of other measures that will serve as covariates in the analysis, such as patients’ health status, memory ability, state anxiety, state depression, and medical knowledge. The rationale for including these measures is that they are potential moderators of participants’ recall of discharge information and thus should be included in the statistical analysis.

## Safety Outcomes

NA

# STUDY DESIGN

## General study design and justification of design

The study will use a cluster randomized controlled trial. Clusters of physicians (cohorts of six physicians commencing training in a three-month period; total of four clusters in the one-year intervention period) will be assigned to one of two types of training (information-structuring skills vs. emotion-handling skills). The physicians won’t know to which cluster they belong. The primary outcome of the study will be patients’ recall of information as a function of physicians’ training. For this purpose, we aim to recruit 50 patients presenting to the emergency department with chest pain and 50 patients with abdominal pain in each three-month period (*N* = 400, in the one-year study period) and document the respective discharge communication (via audio recordings). In addition, each patient will be asked to recall the discharge information immediately after the discharge event, seven days and 30 days later (telephone interview), as well as complete a number of other measures to assess secondary outcomes, such as overall satisfaction and adherence to recommendations. The study will be conducted at the emergency department of the University Hospital of Basel (Switzerland).

## Methods of minimising bias

### Randomisation

Clusters of physicians will be pseudo-randomly assigned to one of the two training programs (information structuring vs. emotion-handling skills training).

Table 3. Training/Interviewing Timeline for Each Cluster of Physicians (*k*)/Patients (*n*)

| Cluster | Months 1-3 | Months 4-6 | Months 7-9 | Months 10-12 |
| --- | --- | --- | --- | --- |
| 1 | Information Structuring  (*k*=6, *n* = 100) |  |  |  |
| 2 |  | Emotion Skills  (*k*=6, *n* = 100) |  |  |
| 3 |  |  | Information Structuring  (*k*=6, *n* = 100) |  |
| 4 |  |  |  | Emotion Skills  (*k*=6, *n* = 100) |

### Blinding procedures

Physicians at the University Hospital Basel will receive a communication skills training (information structuring or emotional skills training) without knowing about the other training and are therefore blinded.

### Other methods of minimising bias

The two trainings will follow the same structure, with physicians receiving the same intensity of teaching. Both programs will consist of one four-hour session of training with an experienced communication expert (Prof Dr Wolf Langewitz). Also, both training programs will consist of theory and practice components as well as a test session to assess the actual use of communication skills (see Table 4).

Table 4. Outline of Physician’s Information Structuring and Emotion Skills Training

| Training | Module | Description |
| --- | --- | --- |
| Information Structuring | Theory (1h) | 1. Rationale for communication training  2. Rationale for information structuring training  3. Introduction to InFARCt scheme |
|  | Practice (3h) | 1. Instructor example 2. Practice in dyads 3. Overall feedback |
|  | Test (5 min per trainee) | 4. Vignette plus information delivery |
| Emotion Skills | Theory (1h) | 1. Rationale for communication training  2. Rationale for emotion skills training  3. Introduction to NURSE scheme |
|  | Practice (3h) | 1. Instructor example 2. Practice in dyads 3. Overall feedback |
|  | Test (5 min per trainee) | 4. Vignette plus information delivery |

## Unblinding Procedures (Code break)

We will not need unblinding. The physicians and experimenters will have full knowledge of the treatment underway and patients will be blind to the condition throughout. Only the data analysis/transcription of discharge and phone interviews will be done in a blind fashion because participants will receive some code (e.g., random alphanumeric code) to prevent identification by the researcher.

# STUDY POPULATION

## Eligibility criteria

Inclusion:

We will train a total of 24 physicians. The intervention will be assessed by interviewing a total of 400 patients who present to the emergency department of the University Hospital Basel with chest pain or abdominal pain and who are seen by one of the study participants.

We will use the following exclusion criteria concerning patients with chest pain (see Figure 3):

- Patients *younger than 18 years of age* will not be recruited because of limited ability to provide informed consent.
- Patients with *limited ability to communicate in German* (the default language at the hospital) will not be recruited to exclude additional confounds related to language proficiency.
- Patients with *dementia* will not be recruited to avoid additional confounds arising from pathological memory deficits.
- Patients with *increased troponin level*, an indicator of myocardial infarction, will not be recruited to minimize stress on these more vulnerable patients.
- Patients with none of five *cardiovascular risk factors* (cvRF; smoking history, diabetes, hypertension, dyslipidemia, age above 50 years, or family history of coronary heart disease) will not be recruited because chest pain symptoms in such patients are typically indicative of diagnoses unrelated to coronary heart disease, requiring a very different follow-up and health management recommendations (e.g., in the case of an anxiety disorder).

*
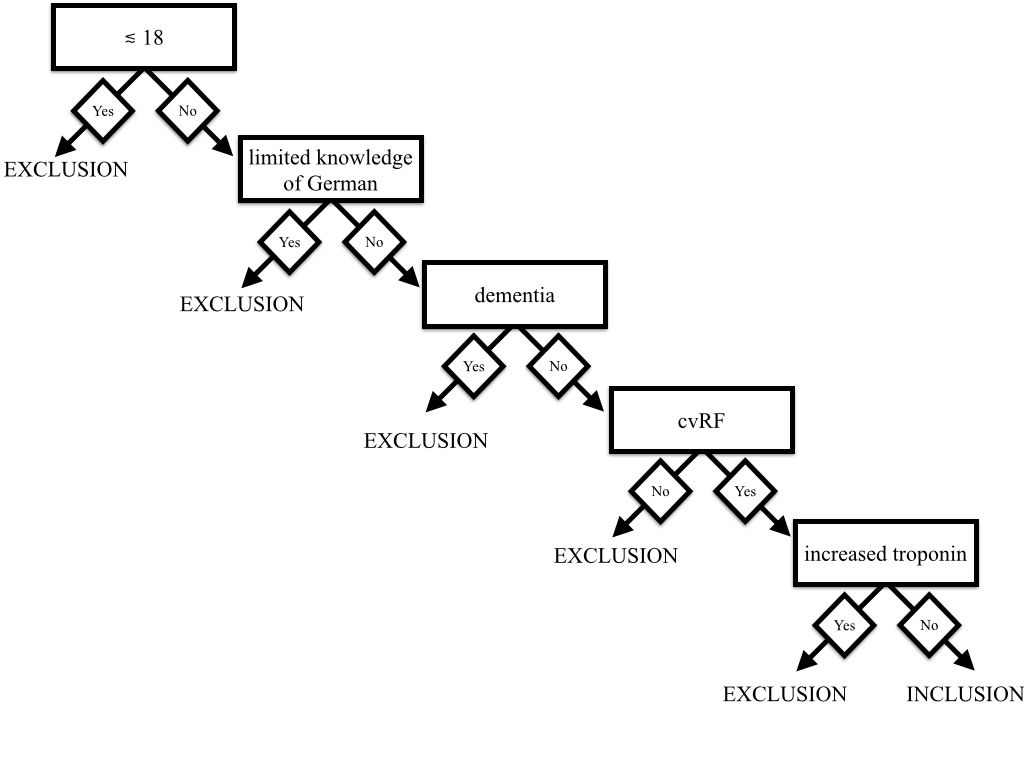
*

Figure 3. Decision Tree for Participant Exclusion/Inclusion in the Intervention Assessment

We will use the following exclusion criteria concerning patients with abdominal pain:

- Patients *younger than 18 years of age* will not be recruited because of limited ability to provide informed consent.
- Patients with *limited ability to communicate in German* (the default language at the hospital) will not be recruited to exclude additional confounds related to language proficiency.
- Patients with *dementia* will not be recruited to avoid additional confounds arising from pathological memory deficits.

## Recruitment and screening

All physicians participating in the study will be asked to contact a member of the study team about any pending discharge communication with a patient suffering from chest pain or a patient suffering from abdominal pain. Eligible participants (see 7.1) will be asked to give written consent to participate in the study. There will be no monetary compensation for the participation.

## Assignment to study groups

The first communication skills training will be randomly assigned by *randomizer.org*. After knowing which communication skills training will be the first, the trainings will be alternately assigned.

## Criteria for withdrawal / discontinuation of participants

If participants withdraw their informed consent, are non-compliant or suffer from unexpected health problems they will be withdrawn from the study. If the investigator or a physician have concerns about safety issues the participant will be withdrawn from the study as well.

# STUDY INTERVENTION

## Identity of Investigational Products (treatment / medical device)

We aim to examine the advantages of an information-structuring skills training for physicians (compared to an emotion skills training) on discharge communication and associated patient outcomes.

### Experimental Intervention (treatment / medical device)

Communication skills training: information structuring skills training

### Control Intervention (standard/routine/comparator treatment / medical device)

Communication skills training: emotional skills training

### Packaging, Labelling and Supply (re-supply)

NA

### Storage Conditions

NA

## Administration of experimental and control interventions

### Experimental Intervention

Communication skills training: information structuring skills training

### Control Intervention

Communication skills training: emotional skills training

## Dose / Device modifications

NA

## Compliance with study intervention

NA

## Data Collection and Follow-up for withdrawn participants

Patients will be excluded if informed consent is withdrawn or the patient doesn’t participate in follow-ups.

## Trial specific preventive measures

NA. This is a study on communication.

## Concomitant Interventions (treatments)

No treatments.

## Study Drug / Medical Device Accountability

NA

## Return or Destruction of Study Drug / Medical Device

NA

# STUDY ASSESSMENTS

## Study flow chart(s) / table of study procedures and assessments

Eligible participants (see section on Participants above) will be asked to give written consent to participate in the study and complete a pre-discharge assessment. The pre-discharge assessment will take about 15 minutes and aims to collect basic demographic measures concerning each patient as well as a number of other measures that will serve as covariates in the analysis, such as patients’ health status, memory ability, state anxiety, state depression, and medical knowledge.

Immediately after the discharge communication event, patients will be interviewed for up to five minutes to assess recall of information provided during the discharge consultation and to assess patients’ satisfaction with the encounter.

Finally, two brief interviews of 5–10 minutes will be conducted over the phone seven days after discharge and 30 days after discharge to retest recall of discharge information, thus assessing the reliability of the main outcome measure and the time component of recall.

For more information see also section “study schedule”.

## Assessments of outcomes

The pre-discharge assessment will take about 15 minutes and aims to collect basic demographic measures concerning each patient as well as a number of other measures that will serve as covariates in the analysis, such as patients’ health status, memory ability, state anxiety, state depression, and medical knowledge. The rationale for including these measures is that they are potential moderators of participants’ recall of discharge information and thus should be included in the statistical analysis.

Immediately after the discharge communication event, patients will be interviewed for up to five minutes to assess recall of information provided during the discharge consultation and to assess patients’ satisfaction with the encounter.

Finally, two brief interviews of 5–10 minutes will be conducted over the phone seven days after discharge and 30 days after discharge to retest recall of discharge information, thus assessing the reliability of the main outcome measure and the time component of recall. In addition, patients will be asked about self-reported adherence to discharge recommendations, and overall assessment of the discharge consultation. Table 5 provides a description of the measures collected in each phase. Patients will also be asked about information provided by their General Practitioners to control for possible confounds of receiving extra or additional information from a third-party.

Table 5. List of Measures Used in Patient Interviews and Respective Description

| Phase | Measure | Description |
| --- | --- | --- |
| Pre-discharge  Assessment | Demographics | Age, Sex, Education, Marital Status, Nationality |
|  | Health-related quality of life | German version of the SF-12,^29^ a generic, short-form health survey with 12 items developed to provide a valid and reliable method to monitor subjective aspects of health and quality of life. |
|  | Memory | CERAD Word List Memory test, a free recall memory  test that assesses learning ability for new verbal information.^30^ A ten-item word list is presented over three trials (at the rate of 1 every 2 seconds) with a different word order each trial. The participant is instructed to read each word aloud as it is presented and then asked to recall as many words as possible. |
|  | State anxiety and depression | German version of the Hospital Anxiety and Depression Scale (HADS-D),^31^ a brief instrument containing two scales of 7 items assessing state anxiety and depression. |
|  | Medical knowledge | Multiple-choice test consisting of 6 questions of increasing difficulty developed by our research team. |
| Interview | Immediate recall | Patients will be asked to freely recall all information provided immediately after the discharge event, “Can you tell me all the information your physician just provided?”, and repeated use of prompts, “Was there any other information you can recall?” until the patient cannot recall any more information. |
|  | Satisfaction | Once patients cannot recall any additional information, they will be asked to characterize their satisfaction with the discharge communication event and physician. Specifically, we will previously developed items (see Study 4) asking patients to rate on a scale from 1 to 10 the (1) physician’s comprehensibleness, (2) information structuring, and (3) willingness to recommend the physician to family and friends. |
| Telephone Interview | Delayed recall | Patients will be asked to freely recall all information mentioned in the discharge event, “Can you tell me all the information your physician provided when you left the emergency department?”, and repeated use of prompts, “Was there any other information you can recall?” until the patient cannot recall any more information. In addition, patients will be asked whether they have received additional information from their general practitioner since discharge. |
|  | Adherence to recommendations | Patients will be asked to list all the physician recommendations that they have actively pursed, “Can you tell me which of your physician’s recommendations you have pursued?”. |
|  | Overall assessment | German version of the Patient Reactions Assessment (PRA-D),^32^ composed of three 5-item scales designed to measure information, affective, and communication components of physician–patient communication. |

### Assessment of primary outcome

The primary outcome of the study will be patients’ recall of information as a function of physicians’ training. For detailed assessment see Table 5.

### Assessment of secondary outcomes

The secondary outcomes will be higher patient satisfaction, better adherence to medication, more adequate disease management, and reduced anxiety. For detailed assessment see Table 5.

### Assessment of other outcomes of interest

Basic demographic measures concerning each patient as well as a number of other measures that will serve as covariates in the analysis, such as patients’ health status, memory ability, state anxiety, state depression, and medical knowledge. The rationale for including these measures is that they are potential moderators of participants’ recall of discharge information and thus should be included in the statistical analysis. For detailed assessment see Table 5.

### Assessment of safety outcomes

NA

#### Adverse events

NA

#### Laboratory parameters

NA

#### Vital signs

NA

### Assessments in participants who prematurely stop the study

Data from the participants won’t be used for the study. There won’t be any disadvantages for the participant.

## Procedures at each visit

### Pre-discharge assessment

Patients will be asked to participate in the study before they have their discharge event. If they agree to participate they will be asked about they’re demographics health-related quality of life, their memory and about their state anxiety and depression. For detailed assessment see Table 5.

### Interview

After the discharge event they will be asked what they can immediately recall and about their satisfaction with the discharge event and the physician. For detailed assessment see Table 5.

### Telephone or personal interview 1&2

And in the telephone interviews (7 days and 30 days after the discharge event) they will be asked to give a delayed recall, how strictly they adhered to the recommendations and there will be an overall assessment. For detailed assessment see Table 5.

# SAFETY

## Drug studies

NA

### Definition and assessment of (serious) adverse events and other safety related events

NA

### Reporting of serious adverse events (SAE) and other safety related events

NA

### Follow up of (Serious) Adverse Events

NA

## Medical Device Category C studies

NA

### Definition and Assessment of (Serious) Adverse Events and other safety related events

NA

### Reporting of (Serious) Adverse Events and other safety related events

NA

### Follow up of (Serious) Adverse Events

NA

## Medical Device Category A studies

### Definition and Assessment of safety related events

Health hazards that require measures:

There won’t be findings in the trial that may affect the safety of study participants and which require preventive or corrective measures intended to protect the health and safety of study participants.

### Reporting of Safety related events

Reporting to Sponsor-Investigator:

Health hazard that require measures are reported to the Sponsor-Investigator within 24 hours upon becoming aware of the event:

Pregnancies:

Reporting of pregnancies is not necessary.

Reporting to Authorities:

In Category A studies it is the Investigator’s responsibility to report to the local Ethics Committee health hazards that require measures within 2 days.

# STATISTICAL METHODS

## Hypothesis

We hypothesize that patients receiving structured discharge information from their trained physicians will be able to recall more information and show higher adherence to recommendations relative to controls (i.e., patients receiving discharge information from doctors trained in emotion-handling skills). We’ll therefore collect data from 200 patients with chest pain and from 200 patients with abdominal pain during 1 year.

## Determination of Sample Size

Within three months 50 patients with chest pain and 50 patients with abdominal pain will be recruited. Including 4 groups with 100 patients this sums up in 400 patients in one year. Sample of 400 gives acceptable power of .8 for comparison of four groups for medium effect sizes of .4.

## Statistical criteria of termination of trial

We’ll stop collecting data after interviewing 400 patients.

## Planned Analyses

The evaluation of the effects of the two training programs will be conducted using mixed-effects model.^33^ Mixed-effects models are statistical models containing both fixed (e.g., intervention program) and random (e.g., patient) effects and are used in a variety of disciplines including medical and social sciences, where repeated measurements are made on the same statistical units or on clusters of related statistical units. Specifically, our analysis will take into account the fact that physicians belong to particular clusters (see Study Design) and the same physician treats several patients, by clustering physicians by training program and patients by physician. These multivariate analysis methods also allow for simultaneous statistical adjustment for covariates of interest, including patient characteristics, such as age, memory ability, and so on. Similar models will be used to estimate the effect of the training programs on patient satisfaction, and adherence to discharge recommendations.

### Datasets to be analysed, analysis populations

The analysis population will be patients with chest pain and patients with abdominal pain. Evaluation groups will be patients having a discharge communication with a physician trained in structured information skills or physicians trained in emotion skills.

### Primary Analysis

The primary analysis will evaluate the effects of the two trainings. This evaluation will be done in January/February 2016 after data collection is done. A doctoral student and the PI will do the data analysis.

### Secondary Analyses

No secondary analyses are planned.

### Interim analyses

No interim analyses are planned.

### Safety analysis

No safety analyses are planned.

### Deviation(s) from the original statistical plan

Possible deviation(s) from the original statistical plan will be described and justified in protocol and/or in the final report.

## Handling of missing data and drop-outs

If participants withdraw their informed consent, are non-compliant or suffer from unexpected health problems they will be withdrawn from the study. If the investigator or a physician have concerns about safety issues the participant will be withdrawn from the study as well.

# QUALITY ASSURANCE AND CONTROL

## Data handling and record keeping / archiving

All collected data will be stored in a central database and kept in compliance with local legal requirements, a minimum for ten years. All relevant data will be anonymously recorded, password secured, using an electronic database. The participant identifiers will be kept confidential.

### Case Report Forms

Data is recorded with electronic Case Report Forms (e-CRF). For each enrolled study participant, a CRF is maintained. CRFs will be kept current to reflect subject status at each phase during the course of study. Participants will not be identified in the CRF by name or initials and birth date. An appropriate coded identification, in our case a participant number, will be used. Only the principal investigator Roland Bingisser, or the investigators Christoph Nickel and Victoria Siegrist will be authorized to make CRF entries. Every authorised person can be identified by his or her own login.

### Specification of source documents

Source data will be available at the site to document the existence of the study participants. Source data will include the original documents relating to the study, as well as the medical treatment and medical history of the participant. The source document will consist of demographic data, date of discharge communication and date of follow ups, participation in study and Informed Consent Forms, randomisation number. Source data are found at the site.

### Record keeping / archiving

All study data will be archived for a minimum of 10 years after study termination or premature termination of the clinical trial. Location of the storage is on site in a locked room.

## Data management

### Data Management System

Data management will be conducted by the Clinical trial unit (CTU) of the University Hospital Basel using *Oracle* and web based *Secutrial®.*

### Data security, access and back-up

CTU provides backup using the systems of the University Hospital of Basel. The study team and for control reasons the members of the ethical committee will have access.

### Analysis and archiving

*Secutrial®* offers easy data extraction to various formats such as SPSS for further analysis. Database is supervised by CTU.

### Electronic and central data validation

*Secutrial®* offers a possibility to create rules for data validation at data entry.

## Monitoring

Source data/documents are accessible to monitors and questions are answered during monitoring. No external sponsor was given right to monitor data.

## Audits and Inspections

The study documentation and the source data/documents are accessible to auditors/inspectors and questions are answered during inspections. All involved parties must keep the participant data strictly confidential.

## Confidentiality, Data Protection

Access to protocol, dataset, statistical code, etc. during and after the study will only be given to members of the study team and to the EKNZ. The University Hospital of Basel grants permission to publish this protocol if the work is accepted, under the CC BY 4.0 license—which means that it will be freely available online, and any third party would be permitted to access, download, copy, distribute, and use these materials in any way, even commercially, with proper attribution.

## Storage of biological material and related health data

Health related data are stored in *Secutrial®.*

# PUBLICATION AND DISSEMINATION POLICY

Publications in peer-reviewed journals are planned.

# FUNDING AND SUPPORT

## Funding

We applied for a founding from SNF. Until April 2015 funding will be provided by the Emergency Department only.

## Other Support

No other support.

# INSURANCE

NA because this is a category A study.

# REFERENCES

1 Samuels-Kalow ME, Stack AM, Porter SC. Effective discharge communication in the emergency department. Ann Emerg Med. 2012;60(2):152-9.

2 Kripalani S, LeFevre F, Phillips CO, Williams MV, Basaviah P, Baker DW. Deficits in communication and information transfer between hospital-based and primary care physicians: implications for patient safety and continuity of care. JAMA. 2007;297(8):831-41.

3 Villanueva T. Transitioning the patient with acute coronary syndrome from inpatient to primary care. J Hosp Med. 2010;5 Suppl 4:S8-14.

4 Kessels RP. Patients' memory for medical information. J R Soc Med. 2003;96(5):219-22.

5 Cameron C. Patient compliance: recognition of factors involved and suggestions for promoting compliance with therapeutic regimens. J Adv Nurs. 1996;24(2):244-50.

6 Galloway S, Graydon J, Harrison D, Evans-Boyden B, Palmer-Wickham S, Burlein-Hall S, et al. Informational needs of women with a recent diagnosis of breast cancer: development and initial testing of a tool. J Adv Nurs. 1997;25(6):1175-83.

7 Mossman J, Boudioni M, Slevin ML. Cancer information: a cost-effective intervention. Eur J Cancer. 1999;35(11):1587-91.

8 Konkelenberg R, Esterman A. Analysis of emergency department patient profiles. Adelaide, Australia: Department of Human Services; 2003. Available from: https://[www.library.health.sa.gov.au/Portals/0/analysis-of-emergency-department-patient-profiles-2003.doc](http://www.library.health.sa.gov.au/Portals/0/analysis-of-emergency-department-patient-profiles-2003.doc)

9 Reichlin T, Hochholzer W, Bassetti S, Steuer S, Stelzig C, Hartwiger S, et al. Early diagnosis of myocardial infarction with sensitive cardiac troponin assays. N Engl J Med. 2009;361(9):858-67.

10 Jones M, Mountain D. Patient information sheets in emergency care. BMJ. 2009;338:b35.

11 Price JR, Mayou RA, Bass CM, Hames RJ, Sprigings D, Birkhead JS. Developing a rapid access chest pain clinic: qualitative studies of patients' needs and experiences. J Psychosom Res. 2005;59(4):237-46.

12 Sanderson BK, Thompson J, Brown TM, Tucker MJ, Bittner V. Assessing patient recall of discharge instructions for acute myocardial infarction. J Healthc Qual. 2009;31(6):25-33; quiz 4.

13 Watson PW, McKinstry B. A systematic review of interventions to improve recall of medical advice in healthcare consultations. J R Soc Med. 2009; 102: 235-43.

14 Johnson A, Sandford J. Written and verbal information versus verbal information only for patients being discharged from acute hospital settings to home: systematic review. Health Educ Res. 2005; 20(4):423-9.

15 Epstein W. Some conditions of the influence of syntactical structure on learning: grammatical transformation, learning instructions, and "chunking". J Verbal Learning Verbal Behav. 1967;6:415-9.

16 Hattie JA. Visible learning: A synthesis of over 800 meta-analyses relating to achievement. New York: Routledge; 2009.

17 Miller GA. The magical number seven, plus or minus two: some limits on our capacity for processing information. Psychol Rev. 1956;63(2):81-97.

18 Gobet F, Lane PC, Croker S, Cheng PC, Jones G, Oliver I, et al. Chunking mechanisms in human learning. Trends Cogn Sci. 2001;5(6):236-43.

19 Chen Z, Cowan N. Chunk limits and length limits in immediate recall: a reconciliation. J Exp Psychol Learn Mem Cogn. 2005;31(6):1235-49.

20 Li G, Ning N, Ramanathan K, He W, Pan L, Shi L. Behind the magical numbers: hierarchical chunking and the human working memory capacity. Int J Neural Syst. 2013;23(4):1350019.

21 Ackermann S, Bingisser M, Heierle A, Langewitz W, Hertwig R, Bingisser R. Discharge communication in the emergency department: physicians underestimate the time needed. Swiss Med Wkly. 2012;142:0:w13588. doi:10.4414/smw.2012.13588

22 Riesenberg LA, Leitzsch J, Little, BW. Systematic review of handoff mnemonics literature. Am J of Med Qual. 2009;24:196-204.

23 Satterfield JM, Hughes E. Emotion skills training for medical students: a systematic review. Med Educ. 2007;41(10):935-41.

26 Dunlosky J, Rawson KA, Marsh EJ, Nathan MJ, Willingham DT. Improving students' learning with effective learning techniques: promising directions from cognitive and educational psychology. Psychol Sci Public Interest. 2013;14(1):4-58.

27 Haines A, Kuruvilla S, Borchert M. Bridging the implementation gap between knowledge and action for health. Bull World Health Organ.2004;82(10):724-33.

28 Back AL, Arnold RM, Baile WF, Tulsky JA, Fryer-Edwards, K. Approaching difficult communication tasks in oncology. CA Cancer J Clin. 2005;55(3):164-77.

29 Gandek B, Ware JE, Aaronson NK, Apolone G, Bjorner JB, Brazier JE, et al. Cross-validation of item selection and scoring for the SF-12 Health Survey in nine countries: results from the IQOLA Project. International Quality of Life Assessment. J Clin Epidemiol. 1998;51(11):1171-8.

32 Galassi J, Schanberg R, Ware W. The Patient Reactions Assessment: A brief measure of the quality of the patient-provider medical relationship. Psychol Assess. 1992;4(3):346-51.

33 West BT, Welch KB, Galecki AT. Linear mixed models: A practical guide to using statistical software. New York: Chapman & Hall/CRC; 2007.

*Note: The references have the same numbering as in the grant application for the SNF and are therefore not continuously numbered.*
