# Supplementary material for: Does information structuring improve recall of discharge information? A cluster randomized clinical trial
Source: PLoS One. 2021 Oct 18;16(10):e0257656. doi: 10.1371/journal.pone.0257656 (PMC8523048; doi:10.1371/journal.pone.0257656)
Supplement: S1 Table — (DOCX) [file pone.0257656.s004.docx]

**Does Information Structuring Improve Recall of Discharge Information? A Cluster Randomized Clinical Trial**

Victoria Siegrist, Rui Mata, Wolf Langewitz, Heike Gerger, Stephan Furger, Ralph Hertwig, Roland Bingisser

**S1 Table. Coding scheme**

A subset of 10 patients were randomly drawn to compute interrater agreements for the use of the coding scheme: An intra-class correlation ICC^39^ for the rater agreement of the number of utterances identified and recalled and Cohen’s Kappa (κ)^40^ for the rater agreement of the assigned codes were calculated. In total, five patients of the E group (two patients with chest pain and three patients with abdominal pain) and five patients from the S group (three patients with chest pain and two patients with abdominal pain) were used for these analyses. Using the R package irr, a two-way random consistency average model was computed. The agreement with an ICC=0.999 and 95% CI=0.998-0.999 showed excellent reliability for 37 transcripts (10 discharge communications, 10 immediate recalls and 17 follow-up transcripts). Cohen’s Kappa for two raters showed an almost perfect agreement with κ=0.88 and 95% CI=0.86-0.91 for 747 coded utterances.

| **Group** | **ID** | **Cat** | **Description** | **Comments** | **Example** |
| --- | --- | --- | --- | --- | --- |
| **Structure** | **St1** |  | Physician provides a "table of contents" | Assessment based on start of discharge communication | 0 = Not given |
|  |  |  |  |  | 1 = Partly given (with St2: as soon as there is one transition) |
|  | **St2** |  | Physician leads explicitly from one segment of the consultation to another | Assessment based on entire discharge communication | 2 = Completely given (even when the content structure is in an adapted form) |
| **Empathy** | **E1** |  | Reaction when patient shows emotions | Assessment over the entire discharge communication, on basis of audio file | 0 = Physician completely ignores emotion |
|  |  |  |  |  | 1 = Physician responds in some way (noticeable pause, echoing, naming an emotion without waiting for the patient's reaction) |
|  |  |  |  |  | 2 = Physician responds to patient's emotion (naming, understanding, respecting, supporting, exploring) |
|  |  |  |  |  | 3 = Could not be judged because the patient shows no emotion |
|  | **E2** |  | Checks with patient whether his concerns are clear | Assessment over the entire discharge communication | 0 = Not given or only "okay?" |
|  |  |  |  |  | 1 = Enquiry in the course of the dialogue, without asking at the end |
|  |  |  |  |  | 2 = Enquiry at the end of the dialogue |
| **Contents** | **I1** | Contents and heading | **In**formation on diagnosis | Any information connected to the content counts as one utterance | First, we come to the **diagnosis** *(1)*. |
|  | **I2** |  | **F**ollow-up | Any information connected to the content counts as one utterance | Then I will discuss the **next steps** *(1)*. |
|  | **I3** |  | **A**dvice on self-care | Any information connected to the content counts as one utterance | In the following, I will show you **what you can do for your own health** *(1).* |
|  | **I4** |  | **R**ed flags | Any information connected to the content counts as one utterance | Then I would like to point out what the **warning signs** are *(1)* and **how you must react** *(2).* |
|  | **I5** |  | **c**omplete **T**reatment | Any information connected to the content counts as one utterance | Finally we come to the **treatment** *(1)*. |
|  | **I6** |  | **"Table of contents" at the beginning of the discharge communication** | Coded as one utterance | I received **advance information** *(1)* prior to the discharge communication |
| **Engagement** | **Question** |  | Every **question** by patients as an indication of commitment | Every question (during discharge communication) counts as one utterance | **How likely is it that I have a vascular condition?** *(1).* |
|  | **Input** |  | Every **active input** by patients as an indication of commitment | Every input (during discharge communication) counts as one utterance | **I can't keep the appointment. I will be on holiday at the beginning of September** *(1)***.** |
|  |  |  |  |  |  |
| **Adherence** | **Ad** |  | **Recommendations** that were mentioned in discharge communication and put into practice by patients | Every recommendation put into practice counts as one item | I've been **looking after myself** *(1)* and **taken paracetamol** *(2)* and **ibuprofen** *(3)*. |
|  |  |  |  |  | I've been **looking after myself** *(1)*, **taken the medication** *(2)* and **not eaten any fruit** *(3).* |
|  |  |  |  |  |  |
| **False memory** | **In1** |  | **Falsely remembered by patients** | Every utterance that was discussed during discharge communication and falsely remembered by the patient | Physician: Please don't eat any chocolate over the next few days. |
|  |  |  |  |  | Patient: I should **eat less chocolate** *(1).* |
|  | **In2** |  | **Not part of the discharge communication** | Every utterance that was not part of the recorded discharge communication but was highly likely discussed at some other time (e.g., with the nurse) |  |
|  |  |  |  |  |  |
| **Information on diagnosis (In)** | **D1** | Investigation | Patient's **[symptom/complaint]** | Every symptom/complaint counts as one utterance | You came to the emergency department because of **chest pain** *(1).* |
|  | **D2** |  | **[Investigation]** that was carried out | Every investigation (including specification) counts as one utterance | First, we did a **scan of your stomach** *(1)*. And then we carried out a **laboratory analysis of your blood** *(2)* and **urine** *(3).* |
|  |  |  |  |  | **The ultrasound carried out by the radiologist** *(1)*. |
|  | **D3** |  | **[Reason]** for carrying out the investigation | Every reason counts as one utterance | We wanted to see **if your pain was caused by a kidney stone** *(1).* |
|  | **D4** |  | **What** the investigation **showed** | Every finding, including "nothing unusual" and "abnormality", counts as one utterance | We could see that the **signs of inflammation are elevated** *(1)****.*** |
|  |  |  |  |  |  |
|  | **D5** |  | **What** the abnormality/result of the investigation **reveales** | Every possible conclusion counts as one utterance | Blood cells in the urine show that your **urine is not completely clean** *(1)*. |
|  | **D6** |  | **[Details]** of the abnormality/result of the investigation | Every detail counts as one utterance | The size of the kidney stone is **3 mm** *(1).* |
|  | **D7** |  | **Refrained** **from** **[investigation]** ... | Every exempted investigation counts as one utterance | In the end, we decided not to carry out a **CT scan** *(1)*... |
|  | **D8** |  | …because **[reason]** | Every reason counts as one utterance | …because of **exposure to radiation** *(1).* |
|  | **D9** |  | **Opinion** of **[another physician]** sought | Every physician counts as one utterance | I have discussed the results with the **gastroenterologists** *(1).* |
|  | **D10** | Diagnosis | **(Suspected) [diagnosis]** lay or medical term | Every diagnosis counts as one utterance | **Gastritis, also known as gastroenteritis** *(1).* |
|  | **D11** |  | **[Confidence rating]** regarding the (exclusion/secondary/differential) diagnosis | Every confidence rating counts as one utterance (e.g., very, quite, just a suspicion) | We are **not certain** *(1)*, but it could be acute gastroenteritis. |
|  | **D12** |  | **Diagnosis could not be made (yet)**/cause of the complaints could not be identified (yet) | This statement counts as one utterance | Unfortunately we have **not discovered** the cause of your pain *(1)*. |
|  | **D13** |  | **[Exclusion diagnosis]** lay or medical term | Every exclusion diagnosis counts as one utterance | We were able to rule out **appendicitis** *(1)*. |
|  | **D14** |  | **[Secondary diagnosis/additional findings]** lay or medical term | Every secondary diagnosis/additional finding counts as one utterance | We have also seen that you suffer from **low blood pressure**, also known as **hypotension** *(1).* |
|  | **D15** |  | **[Differential diagnosis]** lay or medical term | Every differential diagnosis counts as one utterance | But it could also be that you have **stomach worms** *(1)*. |
|  | **D16** |  | **[Explanation]** of the (exclusion/secondary/differential) diagnosis | Every specification counts as one utterance | Answer to the question: "Is it *acute* gastroenteritis?" "Yes, it is **acute** gastroenteritis" *(1).* |
|  | **D17** |  | [Reason] for this suspicion | Every reason counts as one utterance | Especially since you **were abroad, where you ate out** *(1)*. |
|  | **D18** |  | **Explanation of interrelationships or consequences (physiology, etiology, symptoms, diagnosis)** | Every history counts as one utterance | Because the **mucous membranes of the stomach are no longer protected by the acid, there is a wound and this then causes the pain** *(1)*. |
|  | **D19** |  | **Expected course** of disease | Every pathogenesis counts as one utterance | There may be a **further increase in pain** *(1)* in the next few days. **There should be an improvement** *(2)* in a week at the latest. |
|  |  |  |  |  | Your symptoms should **go away by itself** *(1).* |
|  | **D20** |  | Expected [**timeframe**] of the pathogenesis | Every time designation counts as one utterance | There may be a further increase in pain **in the next few days** *(1)*. There should be an improvement **in a week at the latest** *(2)*. |
|  | **D21** |  | Physician **reassures** the patient | Every reassurance counts as one utterance | Gastroenteritis **is not serious** *(1)*. |
|  |  |  |  |  | **You don't need to worry** *(1).* |
|  | **D22** |  | Physician **discharges patient to home** | This statement counts as one utterance | You can **go home** *(1)*. |
|  | **D23** |  | **Any other information** regarding *Diagnosis* | Any other information regarding *diagnosis* counts as one utterance |  |
| **Follow-up (F)** | **F1** | Appointment | No follow-up appointment necessary | This statement counts as one utterance | In my view, you **don't really need a follow-up appointment** *(1).* |
|  | **F2** |  | **[Symptom(s)/differential diagnosis]** that must still be **clarified** | Every symptom/differential diagnosis counts as one utterance | If it really were **stomach worms** *(1)*, you would have to clarify this with your primary care physician. |
|  | **F3** |  | **[Symptom]** must be carefully **monitored** | Every symptom counts as one utterance | Monitor whether you have **extrasystoles** *(1)* in the near future. |
|  | **F4** |  | **[Circumstances]** under which… | Every instance counts as one utterance | **As soon as there are stones in the sieve** *(1)*, you should make an appointment with your primary care physician. |
|  | **F5** |  | **…[something]** should be done | Every action counts as one utterance | As soon as there are stones in the sieve, **you should make an appointment with your primary care physician** *(1)*. |
|  | **F6** |  | Patient should arrange follow-up appointment with [physician/department] | Every appointment counts as one utterance | **Make an appointment with your primary care physician** *(1)***.** |
|  | **F7** |  | Physician has arranged follow-up appointment with [physician/department] | Every appointment counts as one utterance | **I have made a cardiology appointment for you** *(1)*. |
|  | **F8** |  | **[Patient receives message/call]** for the follow-up appointment | Every message/call counts as one utterance | **Our colleagues in cardiology will get in touch with you by phone** *(1)*. |
|  | **F9** |  | **[Date]** regarding follow-up appointment or contact | Every date counts as one utterance | Our colleagues in cardiology will get in touch with you **in the next few days** *(1)*. |
|  |  |  |  |  | I recommend that you have a follow-up consultation with your primary care physician **next week** *(1)*. |
|  | **F10** |  | **[Details]** about follow-up physician | Every detail counts as one utterance | He is a **specialist in this field** *(1)*. |
|  | **F11** | Investigation | **[Follow-up investigation]** lay or medical term | Every investigation counts as one utterance | I recommend that you have a **stress echo test** *(1)*. |
|  | **F12** |  | **[Explanation** of the investigation**]** | Every explanation counts as one utterance | **This is a ultrasonic investigation of your heart under stress conditions** *(1)***.** |
|  | **F13** |  | **[Explanation] why** the investigation will be made | Every reason counts as one utterance | Because you have **diarrhoea** *(1)* and **abdominal pain** *(2)*, you should have another CT scan. |
|  | **F14** |  | **[Duration]** of the investigation | Every date counts as one utterance | The ultrasound examination lasts for **about half an hour** *(1)*. |
|  | **F15** |  | **[Implication]** if there is a specific investigation result | Every implication counts as one utterance | In the case of a negative result, you'll have to get used to the idea of a **cardiac pacemaker** *(1)*. |
|  | **F16** |  | **Follow-up investigation will not take place at the emergence department…** | This statement counts as one utterance | We exclude only the most dangerous here, therefore **you cannot have the investigation carried out by us** *(1)*. |
|  | **F17** |  | …because **[reason]** | Every reason counts as one utterance | **We exclude only the most dangerous here** *(1)*, therefore you cannot have the investigation carried out by us. |
|  | **F18** |  | **[Points]** that **should be discussed** in follow-up investigation | Every point counts as one utterance | I would also mention to your primary care physician that you are **moving house** *(1)* and you are under **stress** *(2)* . |
|  | **F19** |  | [**Documentation**] that will be sent to [physicians/wards] | Every document to be sent counts as one utterance | We will send the **report** *(1)* to your primary care physician in the next few days. |
|  | **F20** |  | [Documentation] that will be sent to [**physicians/wards**] | Every physician or ward counts as one utterance | We will send the report **to your primary care physician** *(1)* in the next few days. |
|  | **F21** |  | [**Date**] when documents are sent/delivered | Every date counts as one utterance | We will send the report to your primary care physician **in the next few days** *(1)*. |
|  | **F22** |  | **[Investigations/treatment]** that relatives should undertake | Every investigation counts as one utterance | Your **partner must also be treated for worms** *(1).* |
|  | **F23** |  | **Any other information regarding** *Follow-Up* | Any other information regarding *follow-up* counts as one utterance |  |
| **Advice on self-care (A)** | **A1** |  | Patient can't do much (with regard to *self-care*) | This statement counts as one utterance | **There is not a lot/nothing special** *(1)* that you can do. |
|  | **A2** |  | Patient should **take care of him/herself** | This statement counts as one utterance | However, you should **take care of yourself** *(1).* |
|  | **A3** |  | **[more/less/abstain from: specific items of food]** | Every item of food counts as one utterance | Avoid **spicy** *(1)* and **fatty** *(2)* foods. Don't drink **coffee** *(3).* |
|  | **A4** |  | **[more/less/abstain from: physical activity/work]** | Every physical activity/work counts as one utterance | You should **walk a lot** *(1)* and **sit as little as possible** *(2)*. |
|  | **A5** |  | No **[contact with (specific groups of) people]** | Every type of contact counts as one utterance | I would recommend that at you **avoid the visit from your brother** *(1)* until Friday. |
|  | **A6** |  | ...(until) **[date]** | Every date provided counts as one utterance | I would recommend that you avoid the visit from your brother **until Friday** *(1)*. |
|  | **A7** |  | …[**reason**] for this measure | Every reason counts as one utterance | ... because you are still very **contagious** *(1)*. |
|  | **A8** |  | **[under which circumstances]** something should/must be done | Every circumstance counts as one utterance | **When you feel well enough** *(1)*, you can go back to work. |
|  | **A9** |  | **Any other information** regarding S*elf-Care* | Any other information regarding *advice on self-care* counts as one utterance |  |
| **Red flags (R)** | **R1** |  | In **[which case/ciricumstance]** ... | Every circumstance counts as one utterance | **If the pain doesn't go away** *(1)*, you should definitely come back. |
|  |  |  |  |  | If the **painkillers don't help** *(1)* … |
|  | **R2** |  | …should the patient present to **[the emergency department/specific physician]** | Every physician or ward counts as one utterance | …you should **come back to the emergency department** (1). |
|  | **R3** |  | ... should the patient present for **[clarification/intervention]** | Every clarification counts as one utterance | ...for an **ECG** *(1).* |
|  |  |  |  |  | …so that we could **admit you as an inpatient** *(1)*. |
|  | **R4** |  | **[Timeframe]** within or outside which something must be acted upon/done | Every date provided counts as one utterance | …in the **next few days** *(1).* |
|  | **R5** |  | **The emergency department is always open** | This statement counts as one utterance | …you **don't need an appointment, you can just come along** *(1)*. |
|  | **R6** |  | **Any other information regarding Red Flags** | Every Any other information regarding *red flags* counts as one utterance |  |
|  | **C1** | Medication | **Prescription** is issued/handed over | This statement counts as one utterance | I've written you **a prescription** *(1)*. |
|  | **C2** |  | **[Medication]** (generic name, brand, type of medication) | Every medication counts as one utterance | **Triatec is a blood-pressure medication** *(1).* |
|  |  |  |  |  | I prescribe you **metamizole** (1) and **aspirin** (2). |
| **complete Treatment (cT)** | **C3** |  | Patient should **continue to take medication** | This statement counts as one utterance | **Continue** *(1)* to take aspirin. |
|  | **C4** |  | **[Reserve medication]** (generic name, brand, type of medication) | Every reserve medication counts as one utterance | As a reserve, I will **give** you **metamizole** *(1).* |
|  | **C5** |  | **[In which scenario]** the (reserve) medication **should be taken** | Every scenario counts as one utterance | Take the medication**, when the pain gets worse** *(1).* |
|  | **C6** |  | Medication **must also be taken** when **symptoms are no longer present** | This statement counts as one utterance | Take the medication for 14 days, **even when the pain has gone away** *(1)*. |
|  | **C7** |  | How the medication **works [process]** | Every explanation counts as one utterance | Riopsan is a gel that **lines the mucous membranes of the stomach and forms a soothing layer** *(1)*. |
|  | **C8** |  | **[Metaphor]** of the process | Every metaphor counts as one utterance | If you cut yourself and **put a plaster on a wound, it doesn't mean that it heals faster. But it helps prevent a new injury. And it's exactly the same with the gastric mucosa** *(1)*. |
|  | **C9** |  | **Reason why the medication is needed** | Every justification counts as one utterance | Pantoprazole, because from time to time **Voltarol (diclofenac sodium) attacks the stomach** *(1).* |
|  | **C10** |  | **[Dose]** | Every dose (e.g., in mg, high/low dose/two at a time) counts as one utterance. | We start with a **dose of 5 mg** *(1).* |
|  | **C11** |  | **How** the medication should be taken | Every explanation (e.g., chewed, swallowed, as a plaster, with food, on an empty stomach) counts as one utterance | Take the medication **on an empty stomach** *(1).* |
|  |  |  |  |  | **Take the tablet with water** *(1).* |
|  | **C12** |  | **How often (max) should medication be taken/temporal distance** | Every frequency (e.g., how often daily or weekly) counts as one utterance | Take paracetamol **four times a day** *(1)*. |
|  | **C13** |  | **[Time (of day)]** when medication should be taken | Every timepoint counts as one utterance | Take paracetamol in the **morning**  *(1)***,** at **lunchtime** *(2)***,** in the **evening** *(3)* and at **night** *(4)*. |
|  |  |  |  |  | **You can already start** *(1)* taking the medication. |
|  | **C14** |  | **Number of** tablets **still to be taken today** | This statement counts as one utterance | That means that you can take **one more tablet today** *(1)*. |
|  | **C15** |  | **[Until when]** should medication be taken | Every day or event counts as one utterance | Take the medication until the **gastric investigation** *(1)*. |
|  | **C16** |  | Which **combinations of medication** are allowed/prohibited | Every explanation counts as one utterance | Do **not take this medication with any other painkiller** *(1).* |
|  | **C17** |  | **[Medication] that the patient should discontinue** | Every medication counts as one utterance | Stop taking the **paracetamol** *(1)*. |
|  | **C18** |  | **[When] the medication should show an effect** | Every timepoint counts as one utterance | The antibiotics should have an **effect from tomorrow** *(1).* |
|  | **C19** |  | **[When] the medication can be collected** | Every timepoint counts as one utterance | You can pick up the medication **tomorrow** *(1).* |
|  | **C20** |  | **[Where] the medication can be collected** | Every place counts as one utterance | There is an **emergency pharmacy** (*1)* here, where you can pick up the medication. |
|  | **C21** |  | The patient has **already received the medication at the emergency department** | This statement counts as one utterance | It's the **same medication that you got here at the emergency department** *(1).* |
|  | **C22** |  | (Possible) **[side effects]** of the medication | Every side effect counts as one utterance | The medication might make you **tired** *(1)*. |
|  | **C23** | Therapy | **[Therapeutic possibility]** | Every possibility counts as one utterance | Kidney stones can be treated **surgically** *(1)* or **conservatively** *(2)*. |
|  | **C24** |  | **[Therapy]** | Every therapy counts as one utterance | For the treatment of musculoskeletal pain, I recommend **physiotherapy** *(1)*. |
|  | **C25** |  | **[Justification]** of need for therapy | Every justification counts as one utterance | Physiotherapy will **strenghten your muscles** *(1) ...* |
|  | **C26** |  | **[Effect]** of the therapy | Every effect counts as one utterance | *...* and this will help you to **have less back pain** *(1)*. |
|  | **C27** | Special | **[Special task]** | Every task counts as one utterance | In the meantime your **urine should be sieved** *(1).* |
|  | **C28** |  | **[Justification]** for special task | Every justification counts as one utterance | The **abdominal belt supports the abdominal wall** *(1)*, which now has holes in it. |
|  | **C29** | Certificate | **Medical certificate** | This statement counts as one utterance | I'll give you a medical certificate... *(1)* |
|  | **C30** |  | **[Duration]** | Date up to which certificate issued counts as one utterance | …for **the next 7 days** *(1).* |
|  | **C31** |  | **Any other information regarding complete Treatment** | Any other information regarding *complete treatment* counts as one utterance |  |
| **Other** | **S1** |  | Advance information about **[who]** has still to see the patient | Every person (e.g., senior physician, nurse) counts as one utterance | Someone **from the study still has to come and see you** *(1).* |
|  |  |  |  |  | A **nurse still has to see you** *(1).* |
|  | **S2** |  | **…**Reason **[why]** this person has still to come | Every reason counts as one utterance | … **to remove the cannula** *(1).* |
|  | **S3** |  | Physician **wishes the patient well/a good recovery** | Every good wish counts as one utterance | **I wish you all the best** *(1)*. |
|  | **S4** |  | **Comment** about **study** | Every comment counts as one utterance | Now we need to **record the consultation** *(1).* |
|  | **S5** |  | **Any other information** | Any other information that is not classified in the "*InFARcT*" and "*other*" groups |  |
