# Supplementary material for: Does information structuring improve recall of discharge information? A cluster randomized clinical trial
Source: PLoS One. 2021 Oct 18;16(10):e0257656. doi: 10.1371/journal.pone.0257656 (PMC8523048; doi:10.1371/journal.pone.0257656)

**Does Information Structuring Improve Recall of Discharge Information? A Cluster Randomized Clinical Trial**

Victoria Siegrist, Rui Mata, Wolf Langewitz, Heike Gerger, Stephan Furger, Ralph Hertwig, Roland Bingisser

**S1 Fig. Visual representation of the raw data**

Discharge communication by group ordered by the number of utterances during discharge


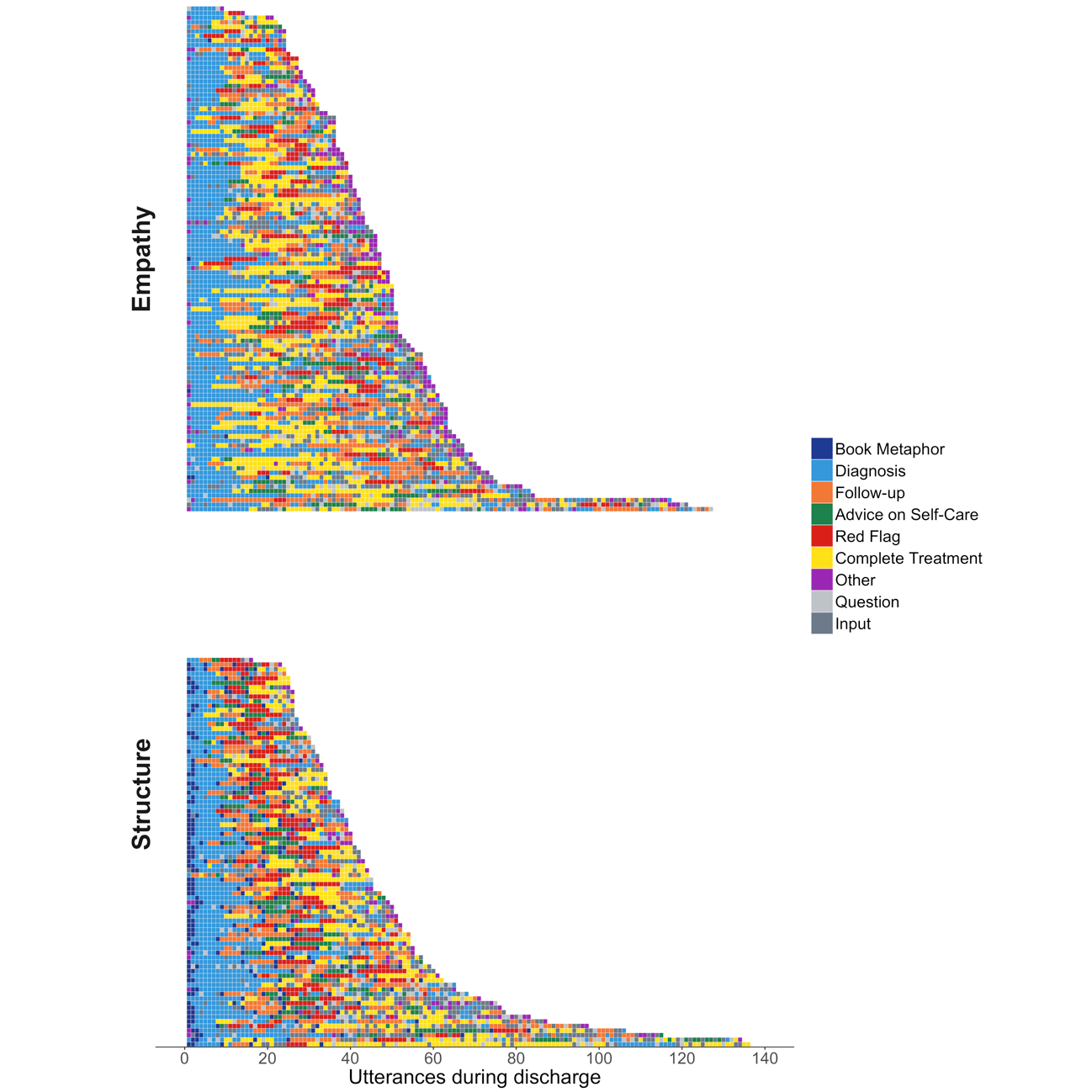


*Note.* Every row represents one discharge communication between the physician and the patient, while every square characterizes one utterance.

Patient’s immediate recall by group ordered by the number of utterances


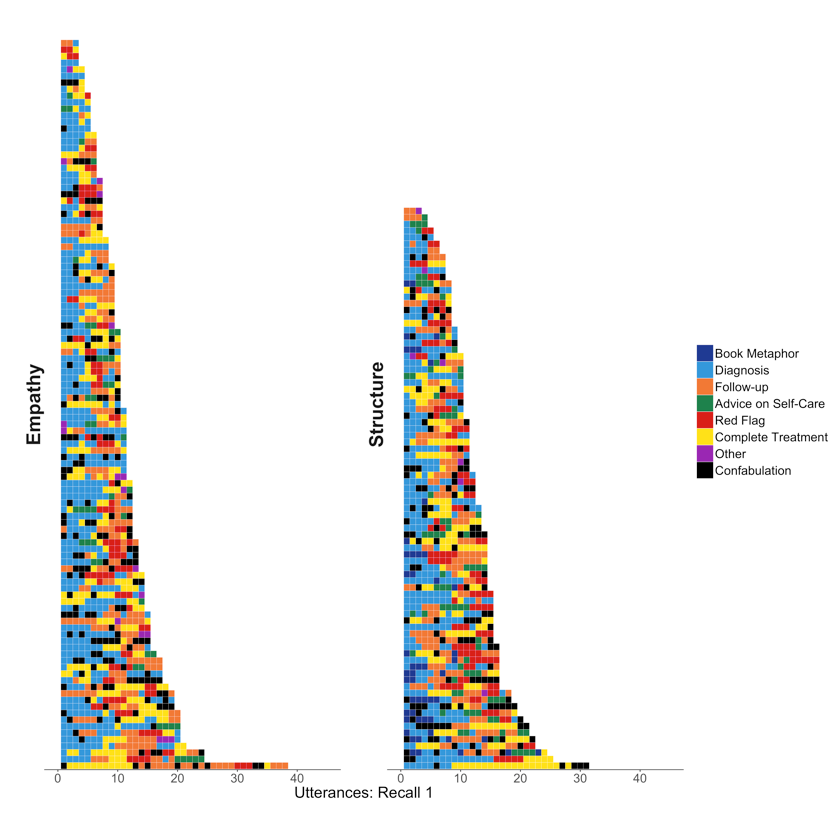


Patient’s recall one week later by group ordered by the number of utterances
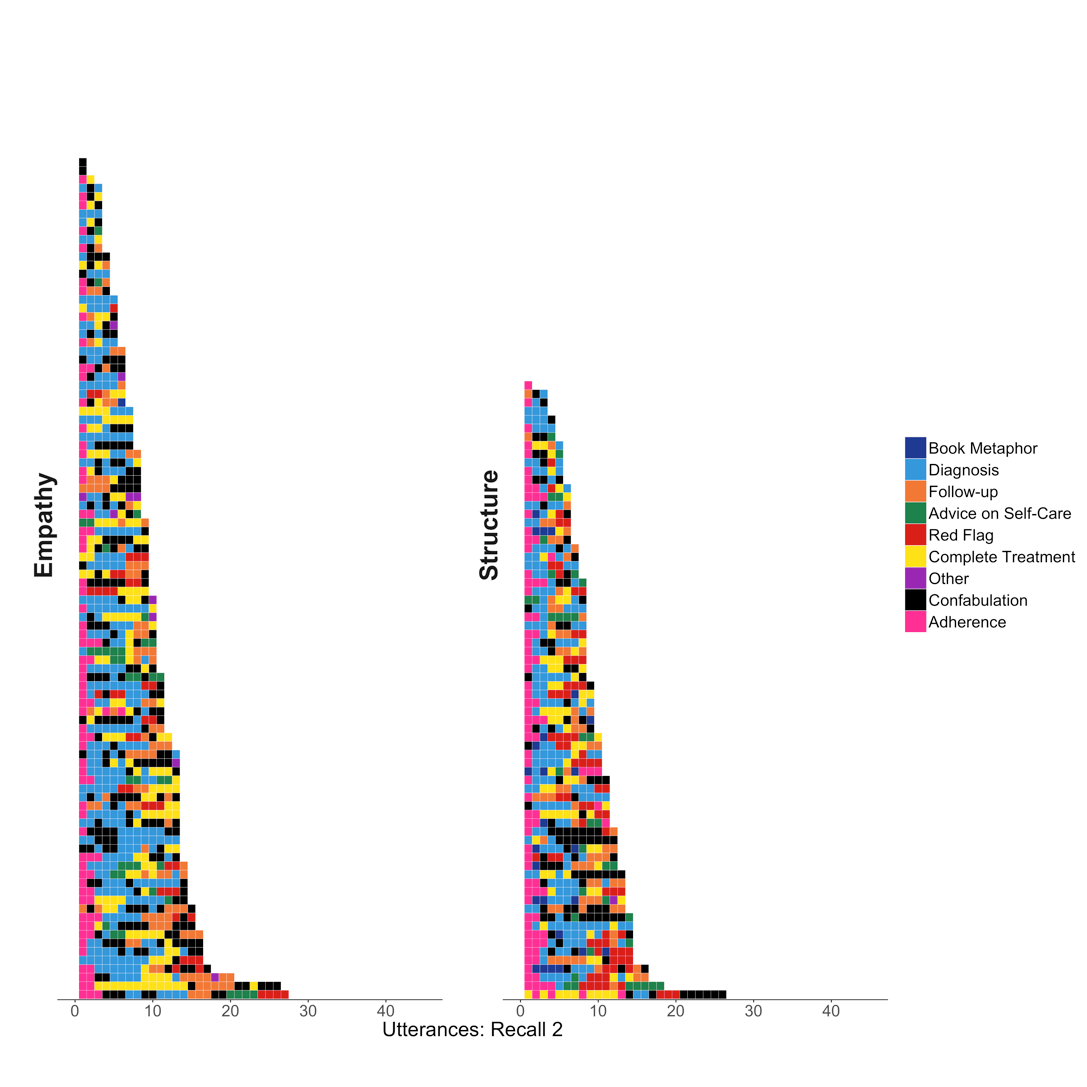


Patient’s recall one month later by group ordered by the number of utterances


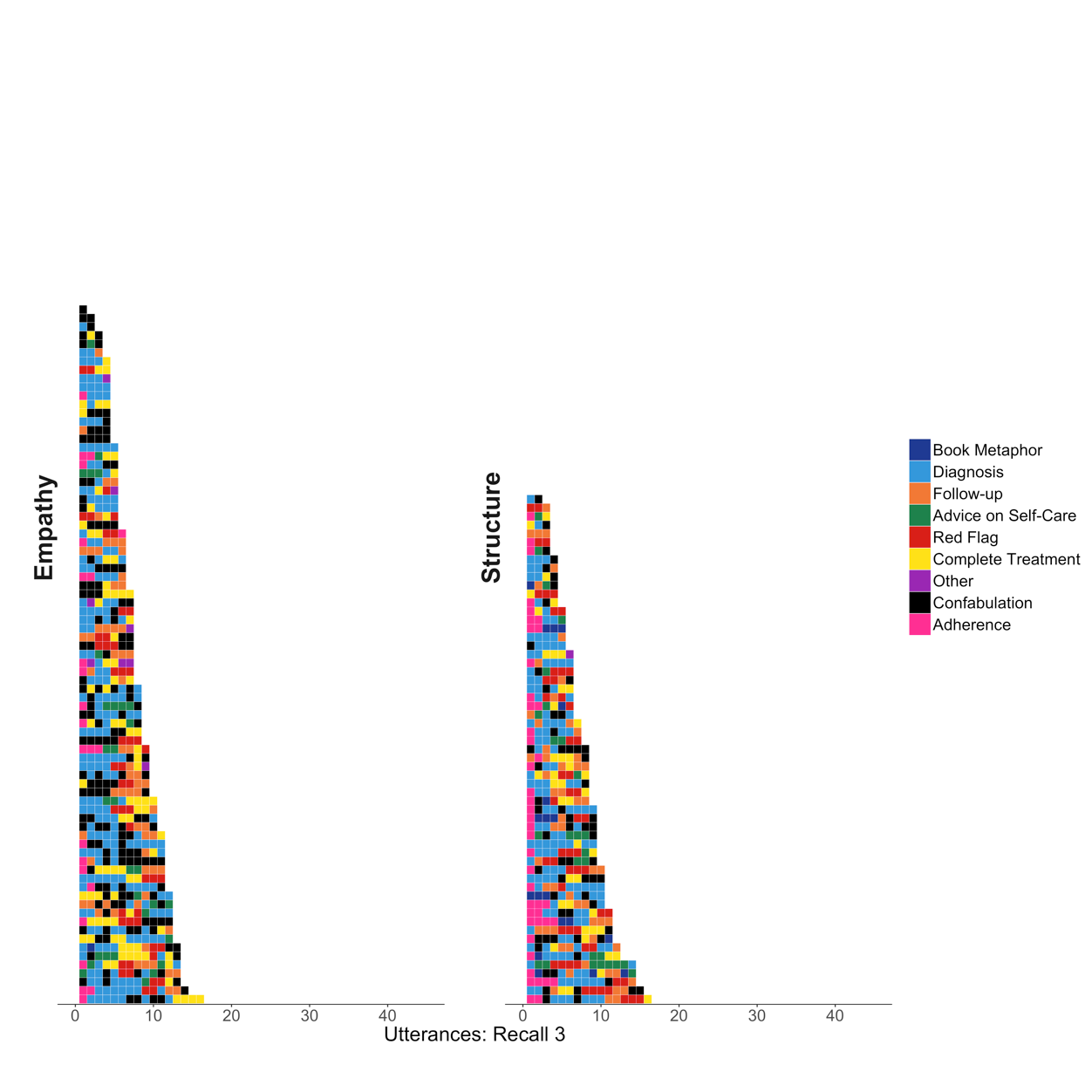

Supplement: S1 Fig — (DOCX) [file pone.0257656.s005.docx]
