# Supplementary material for: Does information structuring improve recall of discharge information? A cluster randomized clinical trial
Source: PLoS One. 2021 Oct 18;16(10):e0257656. doi: 10.1371/journal.pone.0257656 (PMC8523048; doi:10.1371/journal.pone.0257656)
Supplement: S2 Fig — (DOCX) [file pone.0257656.s006.docx]

**Does Information Structuring Improve Recall of Discharge Information? A Cluster Randomized Clinical Trial**

Victoria Siegrist, Rui Mata, Wolf Langewitz, Heike Gerger, Stephan Furger, Ralph Hertwig, Roland Bingisser

**S2 Fig. Visual representation of the raw data from discharge to 30 days follow-up assessment**


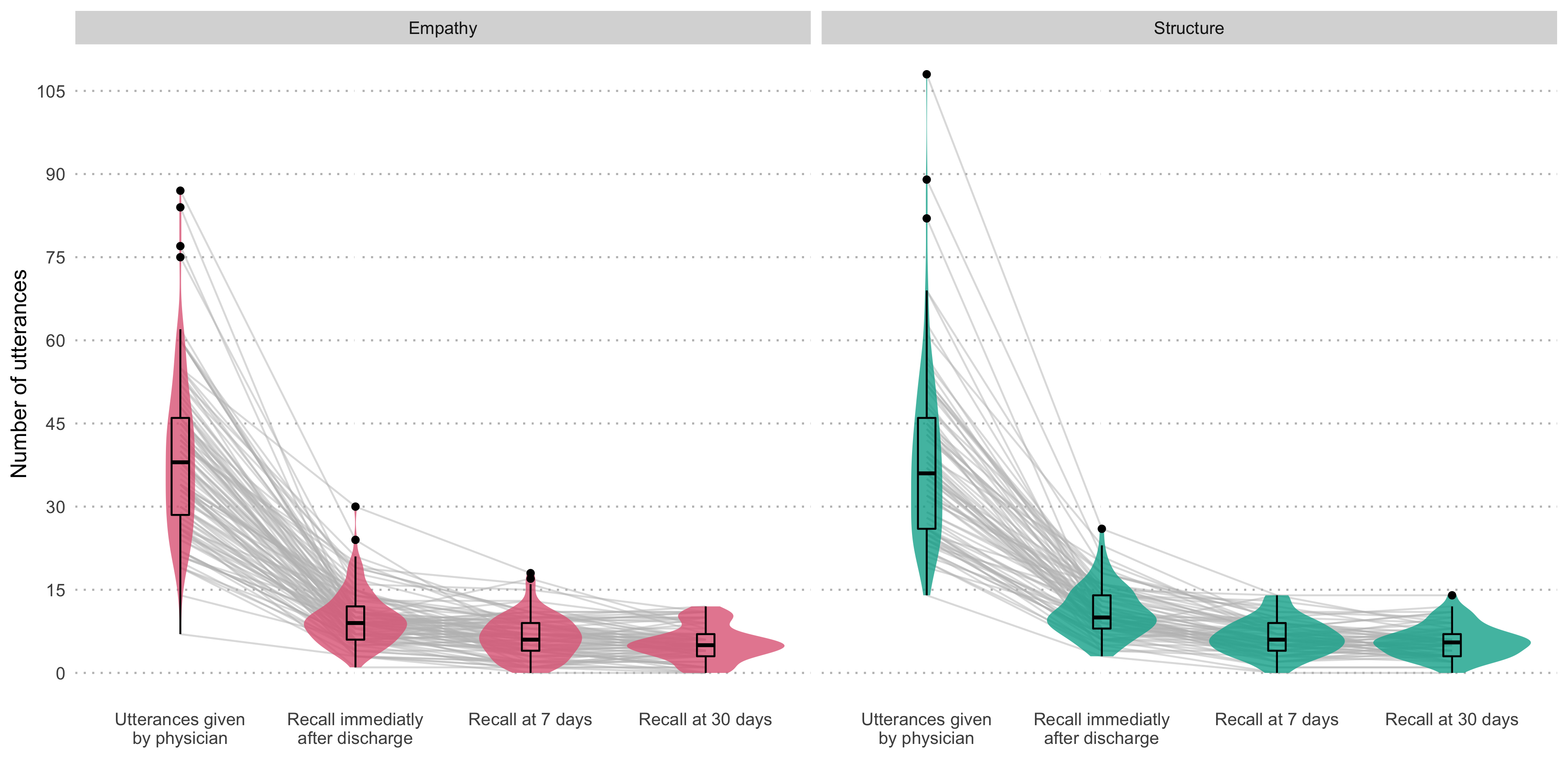


*Note*. Distribution of number of utterances across assessments by groups. The first time point represents the number of utterances given during discharge communication by the physician. The following time points are patient’s recall immediately after discharge, at 7 days, and at 30 days. Grey lines represent individual patient trajectories.
