# Supplementary material for: Does information structuring improve recall of discharge information? A cluster randomized clinical trial
Source: PLoS One. 2021 Oct 18;16(10):e0257656. doi: 10.1371/journal.pone.0257656 (PMC8523048; doi:10.1371/journal.pone.0257656)
Supplement: Attachment — Submitted filename: Rebuttal letter.docx [file pone.0257656.s008.docx]

**Rebuttal letter (PONE-D-20-21645R1)**

**Does Information Structuring Improve Recall of Discharge Information? A Cluster Randomized Clinical Trial**

Editor: Thank you for submitting your manuscript to PLOS ONE. After careful consideration, we feel that it has merit but does not fully meet PLOS ONE’s publication criteria as it currently stands. Therefore, we invite you to submit a revised version of the manuscript that addresses the point raised by Reviewer #2.

**Point-to-point reply to reviewers’ comments:**

**Reviewer #1:**

Thank you for addressing my concerns in this revision. I continue to feel that this is an important contribution. This time through I focused more on the fact that while the training helped with immediate recall, that effect faded over time. This suggests to me that you may want to more directly suggest some follow up structured action to ensure that there is appropriate follow up for ED patients.

Answer: Thank you for your valuable input. We are convinced that the revised version has improved due to your feedback. We have now added a few sentences in the limitations section of the discussion suggesting some follow up structured action to ensure that there is appropriate follow up for ED patients (lines: 507-510):

*“Seventh, though training helped with immediate recall, the waning effect remains to be a concern. We therefore suggest a second structured information as a follow-up to boost recall and support patient adherence. Effects of such an additional structured intervention, however, should be studied in the future.”*

**Reviewer #2:**

The authors have put in great effort to address the comments.

Minor comments

Line 246, 346, the term linear ANOVA to be rephrased to reflect actual test name.

Answer: The term in lines 246 and 346 reflects the actual test name. Please note that Table 2 describes the differences in discharge communication by physicians. We chose linear ANOVA for group comparison as described in the methods section (lines: 245-246). Linear-mixed effects model analyses were used for the primary outcome in patients so that we could control for random factors such as cluster (lines: 249 – 257). Now all table footnotes include the name of the statistical test that was performed to ensure clarity.

With this rebuttal letter and the marked-up copy of our manuscript, we hope to answer all questions raised and would like to thank all reviewers and the editor once more for their valuable input.

Kind regards,
Dr. Victoria Siegrist & Prof. Dr. Roland Bingisser, representing all authors of the manuscript
